# Selective Lithium Recovery via Photothermal Evaporation and Hydration‐Controlled Adsorption

**DOI:** 10.1002/advs.202520066

**Published:** 2026-02-10

**Authors:** Yanan Pan, Yue Zhang, Guoliang Liu, Weiquan Zhan, Wencai Zhang

**Affiliations:** ^1^ Department of Mining and Minerals Engineering Virginia Polytechnic Institute and State University Blacksburg Virginia United States; ^2^ Department of Chemistry Virginia Polytechnic Institute and State University Blacksburg Virginia United States; ^3^ Department of Chemical Engineering Virginia Polytechnic Institute and State University Blacksburg Virginia United States; ^4^ Department of Materials Science and Engineering Virginia Polytechnic Institute and State University Blacksburg Virginia United States; ^5^ Macromolecules Innovation Institute Virginia Polytechnic Institute and State University Blacksburg Virginia United States

**Keywords:** hydration‐controlled adsorption, ion transport dynamics, layered double hydroxides, photothermal evaporation, selective lithium recovery

## Abstract

Solar‐driven evaporation enables scalable, energy‐efficient lithium recovery from saline water, but it is challenged by slow kinetics, low selectivity, and ionic interference. Here, we present a bilayer Mo‐Layered Double Hydroxide@Sponge composite that integrates photothermal evaporation with hydration‐controlled adsorption for selective lithium extraction. A dynamic two‐stage behavior emerges, where photothermal energy initially enhances both ion transport and interfacial water evaporation, followed by hydration‐induced suppression of further adsorption. This transition reflects a shift in the dominant transport regime, where thermal stimulation initially accelerates mobility, but prolonged exposure alters the hydration structure. Elevated temperatures promote the formation of more compact hydration shells around Li^+^, which in turn hinder diffusion by increasing viscosity and enhancing ion‐solvent interactions, highlighting a hydration‐controlled adsorption mechanism. Meanwhile, high salt concentrations contribute to reduced interfacial cohesion, which further facilitates evaporation and reinforces concentration‐driven Li^+^ transport, complementing the hydration‐governed adsorption dynamics. The composite exhibits strong Li^+^ selectivity, structural robustness, and stable solar performance, providing a mechanistic basis for scalable lithium recovery.

## Graphical abstract

A bilayer Mo‐LDH@Sponge composite is developed to couple interfacial photothermal evaporation with hydration‐controlled lithium adsorption. Solar‐induced heating dynamically regulates Li^+^ hydration and transport, enabling selective lithium capture from saline water. This work provides mechanistic insight into photothermal–adsorption coupling for energy‐efficient lithium recovery.

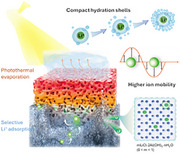

## 1 Introduction

The global community is increasingly confronted with the dual crises of freshwater scarcity and critical metal insecurity, both of which pose significant barriers to sustainable development [1, 2]. Lithium, a cornerstone of modern energy storage systems, is in high demand but suffers from a fragile and geographically limited supply chain [3]. Meanwhile, over 4.3 billion people still lack reliable access to clean water [4]. Interestingly, the Earth's saline resources, such as seawater and salt‐lake brines, contain vast amounts of both water and lithium. These sources represent largely untapped reservoirs, yet they remain difficult to utilize directly due to high salinity and the trace concentration of lithium [5]. If lithium recovery and desalination could be integrated into a single process, it would provide a transformative approach to simultaneously address both resource challenges. However, conventional extraction methods, precipitation, solvent extraction, adsorption, or electrochemical separation, tend to primarily focus on the selective capture of Li^+^ ions, often neglecting the interfacial dynamics of water evaporation and ion transport that occur in saline systems. These approaches are typically energy‐intensive and inefficient, particularly when targeting trace concentrations of lithium in the presence of abundant competing ions.

Three‐dimensional photothermal evaporators have been widely explored for solar desalination, wastewater treatment, and other water purification applications, owing to their large surface area, efficient light absorption, and enhanced vapor transport [6, 7]. However, if their structure were further engineered to possess lithium‐selective absorption sites on the water‐contacting surface, these systems could be transformed into multifunctional platforms for the simultaneous recovery of freshwater and lithium ions. This integrated strategy would allow solar energy to not only drive water evaporation and ion concentration but also enhance lithium uptake through interfacial enrichment.

Realizing such synergistic functionality critically depends on the choice of the lithium‐selective adsorbent. Current research on lithium‐selective adsorbents primarily centers around three material families: manganese‐based materials, titanium‐based molecular sieves, and aluminum‐based layered double hydroxides [8]. Notably, lithium‐aluminum layered double hydroxide (Li/Al‐LDH) has attracted growing attention due to its low dissolution rate, favorable desorption performance under near‐neutral conditions, and excellent structural stability [9]. These materials have already been deployed for lithium recovery from salt lakes in Qinghai and Xinjiang, China. The general chemical formula of Li/Al‐LDH is often written as LiX·mAl(OH)_3_·nH_2_O, where X represents interlayer anions, typically Cl^−^ [10]. In this structure, Al^3+^ ions occupy the octahedral sites within the brucite‐like hydroxide layers. In contrast, Li^+^ ions are primarily located in the interlayer region to balance the charge, often associated with the intercalated anions and water molecules.

In parallel, photothermal materials have gained increasing attention for solar‐driven interfacial water evaporation, enabling passive and scalable desalination through localized heating and vapor transport. Bai et al. reported a natural collagen fiber‐based Janus evaporator (P/S@P‐CFs) featuring a hydrophilic‐hydrophobic bilayer structure and exhibiting strong broadband absorption (>97%) across the wavelength range of 240–2500 nm. This design enabled efficient localized heating, directional vapor transport, and stable evaporation under complex water matrices [11]. The development of efficient photothermal materials has advanced solar evaporation technologies. Among them, molybdenum disulfide (MoS_2_) stands out due to its strong broadband light absorption, high photothermal conversion efficiency, and robust chemical and thermal stability [12]. In addition to its superior photothermal conversion efficiency, MoS_2_ exhibits intrinsic hydrophobicity, which facilitates directional water vapor transport and suppresses salt crystallization on the evaporator surface. This property enhances long‐term operation stability, especially in high‐salinity environments [13, 14]. These attributes make MoS_2_ a strong candidate not only for efficient solar‐driven interfacial evaporation, but also for integration into hybrid systems that couple evaporation with ion transport and adsorption.

Despite advances in both lithium adsorption and photothermal evaporation, most existing systems remain functionally isolated. This separation limits resource utilization efficiency and overlooks key interfacial phenomena that could enhance ion selectivity and mass transfer. The challenge is further exacerbated in high‐salinity, multicomponent environments, where elevated ionic strength and competing cations undermine both adsorption kinetics and stability. These limitations raise critical questions: How can solar energy be harnessed to simultaneously drive interfacial evaporation and directional ion transport? Can a structurally integrated platform combine photothermal conversion with lithium selectivity? And how can such a system maintain performance under realistic aqueous conditions? Addressing these questions requires interface engineering strategies that couple energy input with ion hydration and transport regulation.

To tackle these challenges, we develop a dual‐functional Mo‐LDH@Sponge composite that integrates a MoS_2_‐based photothermal layer, a Li/Al‐LDH adsorption layer, and a porous scaffold for promote efficient interfacial coupling. Under solar illumination, the material simultaneously drives water evaporation and lithium adsorption, where photothermal heating accelerates both solute enrichment and ion transport. We further propose a new metric, termed evaporation‐assisted adsorption gain (EAG), to quantitatively evaluate this synergistic effect, and employ molecular dynamics simulations to elucidate the underlying hydration‐controlled adsorption mechanism. The results reveal a temperature‐dependent evolution of Li^+^ hydration shells, which governs ion mobility and interfacial adsorption dynamics. Collectively, this work establishes a general framework for coupling solar energy harvesting with selective ion separation in saline environments.

## 2 Results and Discussion

### 2.1 Tailored Structure and Properties of Mo‐LDH@Sponge Composite

**FIGURE 1:**
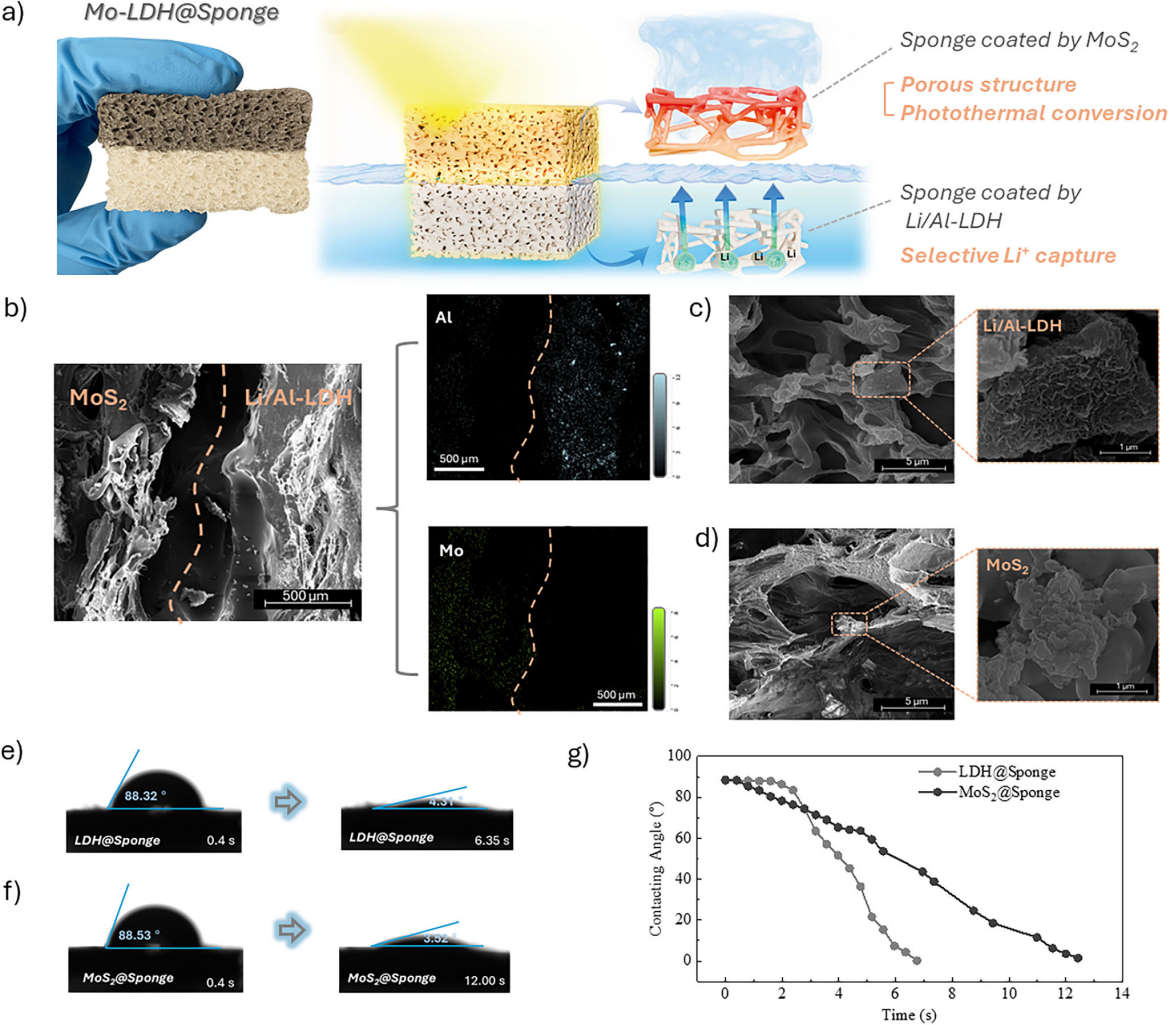
Structural characterization and surface wettability of the Mo‐LDH@Sponge material: (a) Schematic diagram of the bilayer architecture with the top MoS_2_‐coated layer (dark) and bottom Li/Al‐LDH‐coated layer (light); (b) Cross‐sectional SEM image and corresponding EDS elemental mappings of Al and Mo; (c,d) High‐magnification SEM images showing the surface morphology of the Li/Al‐LDH and MoS_2_ layer; (e,f) Water contact angle and spreading behavior; and (g) dynamic contact angle of single‐layer LDH@Sponge and MoS_2_@Sponge materials over time.

To enable simultaneous photothermal desalination and lithium extraction, a bilayer composite, Mo‐LDH@Sponge, was rationally designed and fabricated. As illustrated in Figure 1a, this structure integrates a photothermal MoS_2_ top layer and a lithium‐selective Li/Al‐LDH bottom layer within a porous sponge matrix. The MoS_2_ layer efficiently harvests solar energy and converts it into heat, thereby enhancing water evaporation and facilitating salt removal. In parallel, the Li/Al‐LDH layer selectively captures Li^+^ ions from saline water. This dual‐function configuration presents a simple yet effective platform for integrated solar‐driven desalination and lithium recovery. A schematic 3D video illustrating the process is given in the Supporting Information. Figure 1b presents the cross‐sectional SEM image of the bifunctional Mo‐LDH@Sponge composite. The left region corresponds to the MoS_2_ layer, which exhibits a loose and porous morphology favorable for light absorption and localized photothermal heating. In contrast, the right region represents the Li/Al‐LDH layer, characterized by a denser and more compact surface structure. Elemental mapping of Al and Mo confirms their distinct and well‐defined spatial distributions, indicating that the two functional components were successfully immobilized on opposite sides of the sponge scaffold via impregnation and physical adhesion, thereby forming a clear bilayer interface. High‐magnification SEM images in Figure 1c,d further reveal the microstructural features of each functional layer. The Li/Al‐LDH region displays a uniform, densely packed lamellar structure attached to the sponge skeleton, providing a large number of active sites for Li^+^ adsorption. In comparison, the MoS_2_ region shows a characteristic flower‐like nanosheet morphology with a rougher surface, which enhances light harvesting and promotes efficient photothermal conversion. The morphological contrast and uniform spatial separation between the two layers confirm the successful construction of the bilayer structure and underpin their complementary roles in lithium extraction and solar‐driven water evaporation. Other SEM images, elemental mapping and EDS spectra are given in Figures S1 and S5.

Figure 1e,f further assess the surface wettability of the two functional layers through static contact angle measurements. Both LDH@Sponge and MoS_2_@Sponge exhibit similar initial contact angles of approximately 88°, indicating neutral wettability. However, the wetting kinetics differ markedly: the contact angle of LDH@Sponge drops rapidly to 43.1° within 6.35 s, whereas MoS_2_@Sponge requires 12 s to decrease to 39.2°. This distinction is more clearly illustrated in the dynamic contact angle profile shown in Figure 1g, where LDH@Sponge reaches complete wetting in approximately 6 s, while MoS_2_@Sponge demonstrates a significantly slower wetting process. These results suggest that the LDH layer possesses superior hydrophilicity and faster water uptake, which facilitates more efficient interfacial ion exchange and supports its role in enhancing lithium adsorption performance.

**FIGURE 2:**
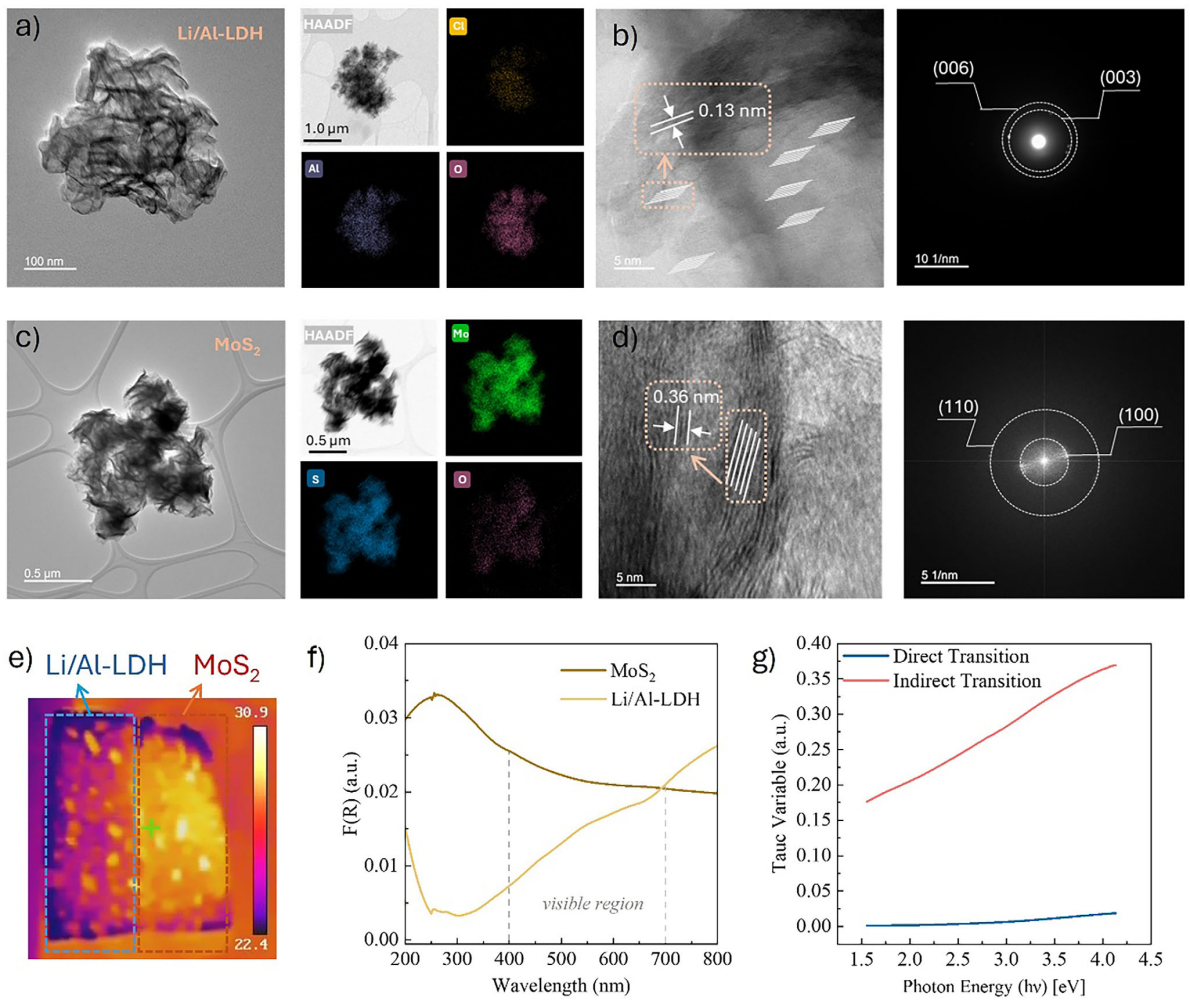
(a) TEM and EDS mapping, (b) HRTEM and SAED of Li/Al‐LDH; (c) TEM and EDS mapping, (d) HRTEM and SAED of MoS_2_; (e) IR thermal image of Mo‐LDH@Sponge under 400 W solar simulator; (f) UV‐vis‐NIR spectra of MoS_2_ and Li/Al‐LDH; (g) Tauc plots of MoS_2_.

Moreover, Figure 2a,b show that Li/Al‐LDH exhibits a typical lamellar stacking morphology with curled edges [15]. Elemental mapping confirms the uniform distribution of Cl, Al, and O, where Al and O form the LDH framework and Cl acts as the interlayer anion. HRTEM reveals a lattice spacing of ∼0.13 nm, and the SAED pattern displays clear (003) and (006) planes, indicating good crystalline and layered structure [16]. In contrast, Figure 2c,d illustrate that MoS_2_ has a flower‐like nanosheet structure with loosely stacked layers, which enhances surface area and photothermal properties [17]. Mo and S are evenly distributed, and the observed lattice spacing of 0.36 nm corresponds to the (002) plane. The Selected Area Electron Diffraction (SAED) pattern shows (100) and (110) rings, confirming the hexagonal crystal phase [18]. These results confirm the structural integrity and functional complementarity of the two materials, supporting their integration into a bifunctional composite for synergistic lithium extraction and solar‐driven evaporation. The XRD spectra in Figure S6 further verify the presence of well‐defined crystalline phases corresponding to layered MoS_2_ and Li/Al‐LDH, confirming the successful construction of the bilayer Mo‐LDH@Sponge architecture.

As shown in Figure 2e, Infrared (IR) imaging of the Mo‐LDH@Sponge under light irradiation reveals a distinct temperature gradient between the two functional layers. The MoS_2_‐coated region exhibits a markedly higher surface temperature compared to the Li/Al‐LDH side, indicating the superior photothermal conversion efficiency of MoS_2_. This observation aligns well with the TEM results, which showed that MoS_2_ possesses a loosely stacked, flower‐like nanosheet morphology that enhances light absorption and promotes localized heating. In contrast, Li/Al‐LDH displays a more compact, ordered lamellar structure that, while less thermally responsive, offers structural stability and strong selectivity for Li^+^ adsorption. These findings support the synergistic role of the bilayer design in enabling simultaneous photothermal evaporation and lithium recovery.

Figure 2f compares the light absorption properties of the two materials. MoS_2_ exhibits significantly stronger absorbance across the 300–700 nm spectral range, with a pronounced absorption feature in the ultraviolet region (250‐300 nm), indicating its excellent optical response. In contrast, Li/Al‐LDH displays markedly much weaker absorbance, with only a slight increase beyond 700 nm in the red‐light region, and an overall lower Kubelka‐Munk F(R) values than MoS_2_. Figure S2a quantifies this difference: MoS_2_ achieves an integrated absorption area of 6.83 a.u.·nm in the 400–700 nm range, nearly double that of Li/Al‐LDH (3.88 a.u.·nm), confirming its superior solar energy harvesting capacity. These optical advantages position MoS_2_ as a promising candidate for photo‐responsive lithium extraction systems.

Additionally, Figure 2g presents the Tauc plot of MoS_2_, indicating that its primary optical transition corresponds to an indirect bandgap, consistent with the electronic structure of bulk or multilayer MoS_2_. Linear fitting of the indirect transition in the 2.4‐3.0 eV range (Figure S2b) yields an estimated bandgap of approximately 1.67 eV, near the edge of the visible spectrum. This further supports the strong photo‐responsiveness of MoS_2_ under solar illumination and its potential utility in broad‐spectrum photothermal applications.

### 2.2 Evaporation‐Enhanced Lithium Adsorption and Selectivity

**FIGURE 3:**
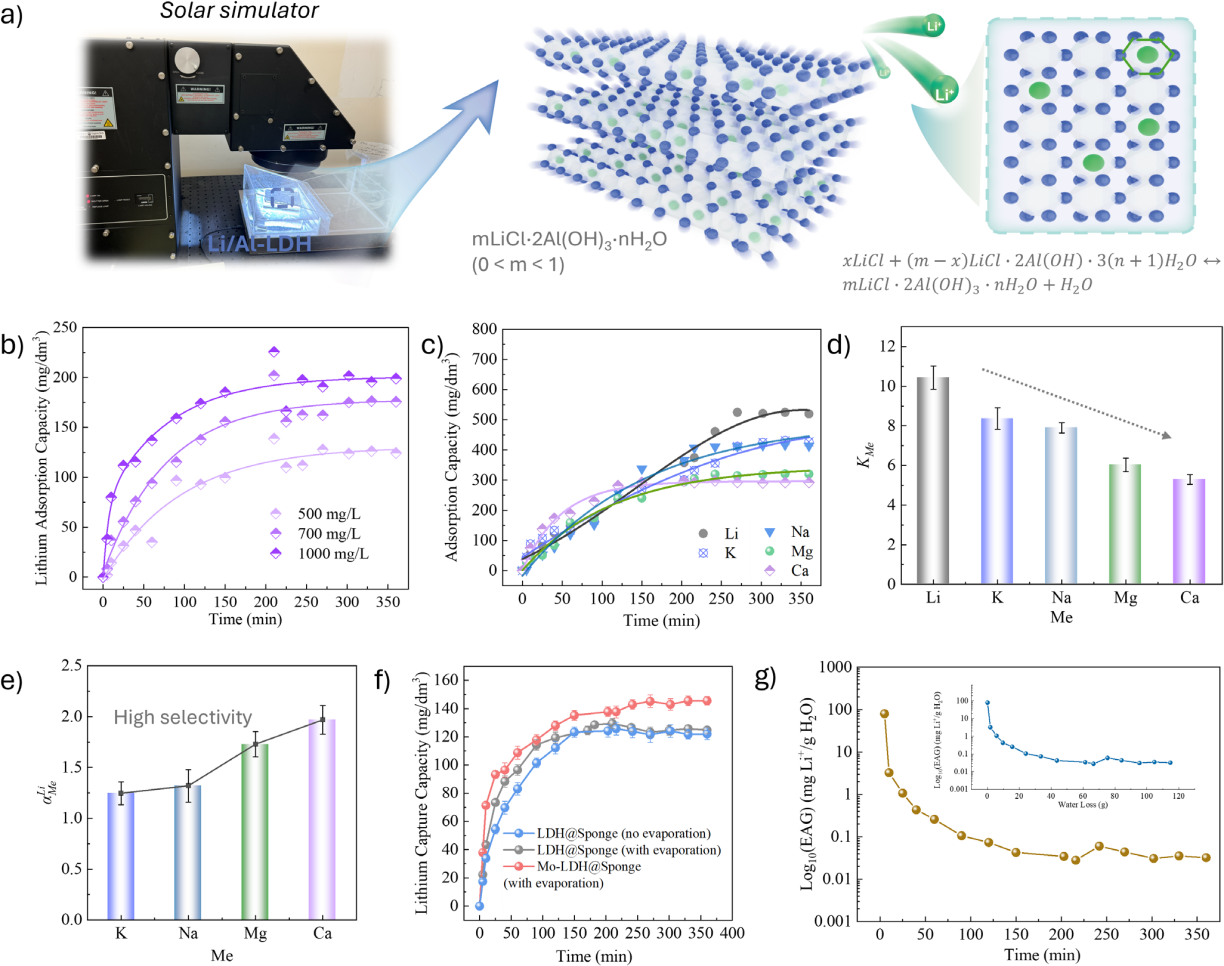
(a) Schematic of the solar simulator setup and Li^+^ intercalation mechanism in Li/Al‐LDH; (b) Lithium adsorption kinetics at different initial concentrations (500, 700, and 1000 mg/L); (c) Adsorption of Li^+^ and other competing cations (Na^+^, K^+^, Mg^2+^, Ca^2+^) in the mixed‐ion system; (d) separation factors and (e) selectivity coefficients of different ions; (f) Li^+^ capture capacity of different materials over time under various conditions; (g) Time‐dependent evolution of EAG plotted on a log10 scale and relationship between EAG and cumulative water loss, shown on a log10 scale.

To evaluate the lithium adsorption performance of the Mo‐LDH@Sponge, the composite was placed in a reaction vessel (Figure 3a) and irradiated under simulated solar illumination. Owing to its strong hydrophilicity, the lower portion of the LDH@Sponge remained fully immersed in the solution, while the upper portion was exposed to air and light. In this configuration, the LDH layer provides abundant active sites for Li^+^ capture. Upon contact with the solution, Li^+^ ions migrate toward and preferentially occupy Al‐O vacancies within the LDH interlayers, driven by the concentration gradient, thereby enabling selective lithium adsorption [19]. The effect of initial Li^+^ concentration on the adsorption kinetics was first investigated. As shown in Figure 3b, Mo‐LDH@Sponge demonstrated a rapid lithium uptake within the first 50 min and approached adsorption equilibrium after approximately 200 min. The related concentration change is given in Figure S3a. The equilibrium adsorption capacity increased with higher initial Li^+^ concentrations, primarily due to the enhanced mass transfer driving force that promoted faster Li^+^ ion diffusion and interaction with the adsorption sites [20]. To further examine the Li^+^ release and desorption behavior, desorption experiments were conducted using solutions with different initial lithium concentrations (Figure S3c). During the first 10 min, the Li^+^ concentration increased rapidly, followed by a gradual stabilization as the system approached equilibrium. Notably, the total amount of desorbed lithium exhibited an inverse correlation with the initial lithium concentration in the desorption solution. This observation suggests that a small amount of Li^+^ present in the desorption medium can reduce the concentration gradient between the material and the solution, thereby mitigating excessive ion release [21].

Adsorption selectivity is a key indicator of a material's separation performance. As shown in Figure 3c, Mo‐LDH@Sponge demonstrates effective adsorption of Li^+^ as well as common competing ions, including K^+^, Na^+^, Mg^2+^, and Ca^2+^. While the material exhibits measurable affinity toward all tested ions, the adsorption capacities for monovalent cations are notably higher than those for divalent cations (Figure S3f). This trend is further supported by the concentration profiles in Figure S3d,e, where the concentrations of all ions decrease progressively over time and remaining water volume, indicating the material's broad‐spectrum adsorption capability.

Figure 3d,e present the separation factors and selectivity coefficients of each ion relative to Li^+^. The results clearly demonstrate that the Mo‐LDH@Sponge exhibits a clear preferential adsorption toward Li^+^ compared with other competing cations, followed by K^+^ and Na^+^, whereas Mg^2+^ and Ca^2+^ show comparatively low affinity toward the adsorbent. Notably, the separation coefficient between Li^+^ and Mg^2^^+^ ($a_{Mg}^{Li}$), which represents a critical selectivity challenge in brine resources, is greater than unity, demonstrating a preferential uptake of Li^+^ even in the presence of divalent competing ions. Similarly, all selectivity coefficients defined relative to Li^+^ exceed 1, confirming a systematic preference for lithium under multi‐ion conditions. This high lithium selectivity is primarily attributed to the structural compatibility between Li^+^ and the Al‐O vacancy sites within the Li/Al‐LDH layers. Due to their larger hydrated radii and stronger electrostatic interactions, other cations‐particularly divalent species‐face greater steric and energetic barriers to intercalation, resulting in reduced adsorption efficiency [22].

To assess the effect of evaporation on Li^+^ adsorption, Figure 3f compares the lithium uptake performance of different material systems under various conditions. In the absence of evaporation, LDH@Sponge exhibits relatively limited lithium adsorption. Upon introducing photothermal evaporation, however, the adsorption capacity increases markedly, indicating that the evaporation process enhances Li^+^ transport and facilitates ion access to adsorption sites. Notably, the Mo‐LDH@Sponge composite‐constructed by integrating LDH@Sponge with an upper MoS_2_@Sponge photothermal layer‐demonstrates consistently superior lithium capture performance throughout the process, reaching a final adsorption capacity of approximately 150 mg/dm^3^. This enhancement underscores the synergistic role of the upper photothermal layer in promoting lithium enrichment and capture within the lower Li/Al‐LDH‐based adsorption region.

**FIGURE 4:**
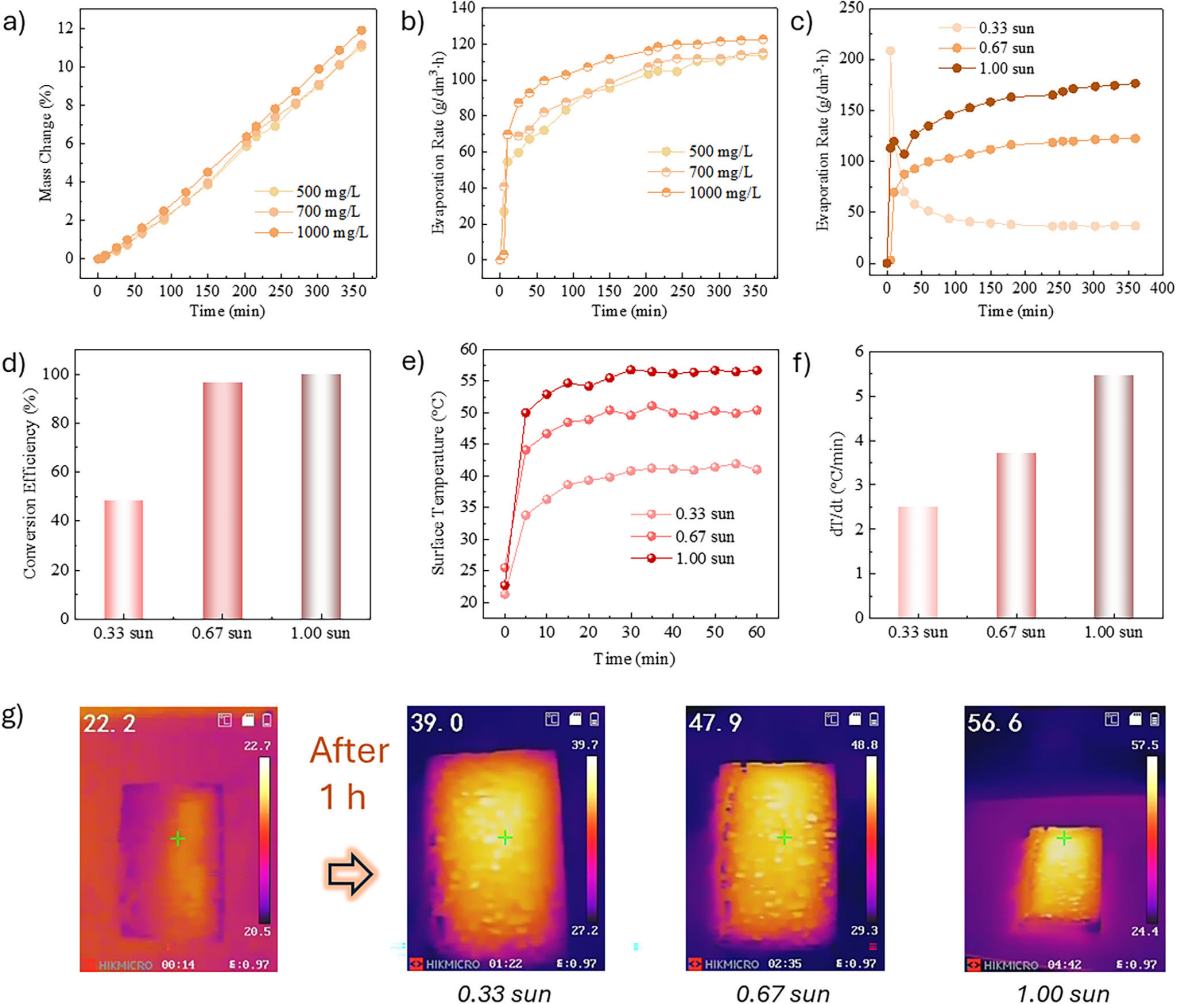
(a) Mass change and (b) evaporation rate over time under various initial Li^+^ concentrations; (c) Evaporation rate over time, (d) Solar‐to‐vapor conversion efficiency, (e) Surface temperature evolution, (f) Heating rate, and (g) IR images of Mo‐LDH@Sponge before and after 1 h of solar illumination under different solar intensities.

To quantitatively evaluate the effect of evaporation on adsorption behavior, Figure 3g depicts the evolution of EAG as a function of reaction time and cumulative water loss, respectively. In both representations, EAG exhibits markedly higher at the early stage, indicating that Li^+^ adsorption per unit of water loss is most efficient when evaporation is rapid (refer to Figure 4b, more details will be discussed in the next section), and Li^+^ ion enrichment is most pronounced. As the reaction proceeds or water loss accumulates, the EAG values gradually decrease and approach a steady state, suggesting a transition toward diffusion‐limited or site‐saturation‐controlled regimes, where the incremental contribution of evaporation to lithium uptake diminishes. These findings reveal that photothermal evaporation plays a critical role in the early stages of adsorption kinetics by increasing local Li^+^ ion concentrations and raising the solution temperature, thereby accelerating mass transfer. In contrast, during the later stages, the effects of solution concentration, and possible surface salt precipitation lead to reduced adsorption efficiency. Therefore, the evaporation‐assisted enhancement is particularly effective in the initial phase of the process. This behavior highlights the underlying mechanism responsible for the superior lithium capture performance observed for Mo‐LDH@Sponge, emphasizing the synergistic advantage of integrating photothermal‐driven evaporation with targeted ion adsorption.

To further contextualize the photothermal‐assisted lithium adsorption performance of the Mo‐LDH@Sponge system, a systematic comparison with representative photothermal‐assisted lithium recovery materials reported in the literature is provided in Table S4. The comparison summarizes key aspects including photothermal components, lithium capture mechanisms, performance metrics, and system‐level advantages and limitations. As shown, previously reported systems predominantly rely on ion‐sieve chemistry, membrane‐based separation, or electrochemical intercalation strategies, whereas the present work integrates photothermal interfacial evaporation with solid adsorption in a structurally unified platform. This comparison highlights the distinct design philosophy and functional integration of the Mo‐LDH@Sponge system among existing photothermal‐assisted lithium recovery approaches.

### 2.3 Photothermal Behavior and Evaporation Performance

To evaluate the photothermal evaporation performance of Mo‐LDH@Sponge, its evaporation behavior was investigated under varying initial Li^+^ concentrations. As shown in Figure 4a,b, the water evaporation rate increased significantly with rising Li^+^ concentration. Besides, Figure 4c depicts the evaporation rate of Mo‐LDH@Sponge over time under different solar intensities. As shown, distinct trends emerge under varying solar intensities. At 0.33 sun and 0.67 sun, the evaporation rates gradually increase and stabilize over time. In contrast, under 1 sun, the evaporation rate rises sharply during the initial stage, reaching a peak within the first 20–25 min, and subsequently shows a slight decline. This phenomenon is likely due to the rapid water loss at high solar intensity, which leads to a reduced liquid‐air interfacial area and regional salt concentration effects near the sponge surface, both of which can suppress further evaporation [23]. These localized changes not only hinder sustained water transport but also create unfavorable conditions for continuous vapor generation. As a result, while higher solar intensity enhances thermal responsiveness, excessively rapid evaporation may induce secondary limitations, such as concentration polarization or surface drying effects (i.e., regional salt concentration and reduced liquid‐air interfacial area), ultimately influencing long‐term evaporation efficiency. Based on the steady‐state mass loss and including both latent and sensible heat contributions, the solar‐to‐vapor conversion efficiency of the Mo‐LDH@Sponge device was determined to be 48.7%, 96.5%, and 100.0% under 0.33, 0.67, and 1 sun illumination (as given in Figure 4d), respectively (see Supporting Information for detailed calculations and area correction). The increase in conversion efficiency with solar intensity is consistent with the enhanced photothermal heating and interfacial evaporation observed above, while the near‐unity efficiency at 1 sun indicates effective solar energy utilization despite the slight decline in evaporation rate at prolonged irradiation, which is attributed to local mass‐transfer limitations such as salt accumulation and reduced liquid–air interfacial area.

To further understand the mechanism behind the solar intensity‐dependent evaporation behavior, the surface thermal response of Mo‐LDH@Sponge was investigated. Figure 4e presents the real‐time surface temperature evolution of Mo‐LDH@Sponge under different solar intensities. A clear correlation is observed between increasing solar intensity and higher surface temperature. This trend is further supported by Figure 4f, which shows that the heating rate increases from approximately 2.4°C/min under 0.33 sun to over 5.0°C/min under 1 sun, indicating enhanced photothermal conversion efficiency at higher intensities. These findings are consistent with the infrared thermal imaging results shown in Figure 4g, where the surface temperature after 1 h of illumination reaches 39.0°C, 47.9°C, and 56.6°C under 0.33 sun, 0.67 sun, and 1 sun, respectively. Collectively, these results highlight the excellent photothermal responsiveness of Mo‐LDH@Sponge and its critical role in promoting rapid heating and efficient evaporation under solar‐driven conditions.

### 2.4 Photothermal‐Modulated Evaporation‐Adsorption Synergy

**FIGURE 5:**
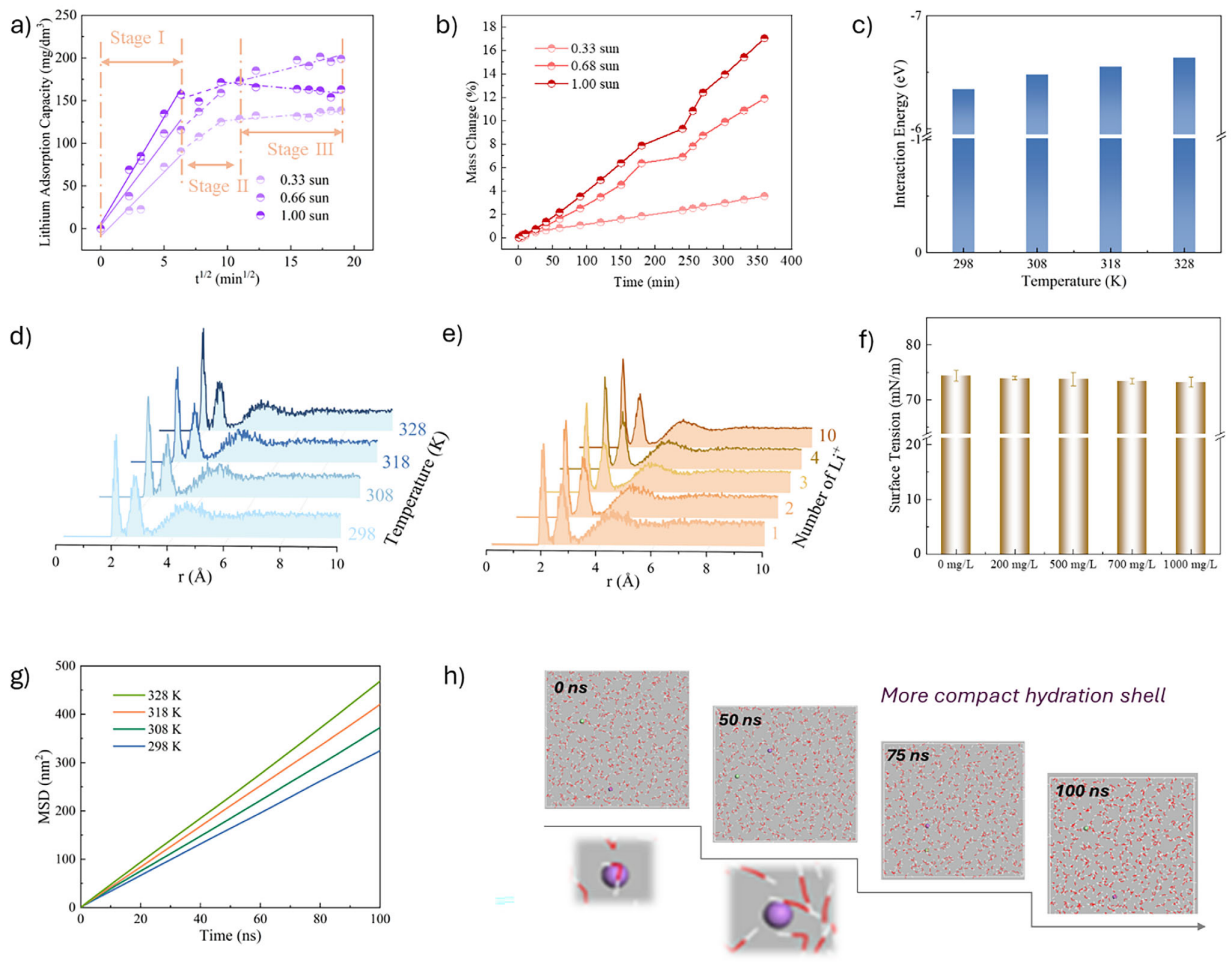
(a) Lithium adsorption kinetics at different solar intensities (0.33, 0.67, 1.00 sun); (b) Mass change over time under different solar intensities; (c) Interaction energies between Li^+^ ions and water molecules; (d) RDF showing the spatial distribution of water molecules around Li^+^ ions under different temperatures; (e) RDF at varying Li^+^ concentrations; (f) Surface tension measurements of different Li^+^ solutions; (g) Mean Square Diffusion (MSD) curves of water molecules at various temperatures; (h) Time‐dependent trajectories of Li^+^ ions and water molecules.

The influence of solar intensity on lithium adsorption kinetics and water evaporation was systematically investigated (Figure 5a,b). To better analyze the effect of solar intensity on the lithium adsorption process, the data were fitted using Weber's intraparticle diffusion model as depicted in following Equation (1):

(1) $$q=k_{id}\;*t^{1/2}+C$$

where *q* is lithium adsorption capacity (mg/dm^3^); *k_id_* is the intraparticle diffusion rate constant (mg/(dm^3 ^⋅min^1/2^)); *t* is the adsorption time (min); *C* is constant.

Based on kinetic fitting, the lithium adsorption process can be divided into three distinct stages, with the corresponding fitting parameters summarized in Table S1. During the initial stage (Stage I), lithium uptake increases steadily with time under illumination, and higher light intensities lead to faster adsorption rates. This behavior can be attributed to photothermal effects that elevate the local temperature, thereby accelerating Li^+^ diffusion and enhancing the surface activity of the LDH layer, which improves site accessibility. A clear shift in adsorption kinetics is observed during Stages II and III, with a pronounced rate maximum occurring at approximately 40 min. Under moderate light intensities (0.33 and 0.67 sun), the adsorption rate continues to increase, whereas at a higher intensity (1 sun) the rate decreases slightly and becomes lower than that observed at 0.67 sun. This trend suggests that adsorption does not reach complete site saturation and that the reduced uptake is not associated with lithium desorption. Indeed, no lithium release is detected under these conditions (Figure S4), indicating that the observed decline in adsorption rate is not driven by Li^+^ desorption. This suggests that excessively high solar intensities may negatively impact adsorption efficiency, potentially due to accelerated water evaporation or changes in surface hydration dynamics. The evaporated portion consists mainly of loosely bound or free water molecules, whereas the tightly coordinated hydration water surrounding Li^+^ ions remains intact. As free water diminishes, the solution becomes enriched in highly hydrated Li^+^ ions, resulting in increased viscosity and decreased ionic mobility. This imbalance, where the Li^+^ diffusion rate cannot keep pace with the rising regional concentration, ultimately impedes further adsorption. Concurrently, the adsorbent approaches saturation, and the system transitions into a mass transfer‐limited regime, as further supported by the Li^+^ concentration profiles in Figure S3b.

To further evaluate the effect of solar intensity on water removal, the photothermal evaporation performance was assessed in Figure 5b. A clear trend was observed: higher solar intensity resulted in significantly greater water loss. This enhancement underscores the role of solar‐driven heating in accelerating interfacial water evaporation and modulating Li^+^ ions mobility. Together, these results highlight a solar‐modulated trade‐off between enhanced adsorption kinetics and water availability. While moderate illumination synergistically boosts both Li^+^ uptake and evaporation, excessive irradiation may hinder ion transport due to the densified hydration effect. These findings emphasize the importance of tuning solar input to optimize the coupled thermal and adsorptive performance of Mo‐LDH@Sponge.

To support the experimental observations and gain molecular‐level insight, molecular dynamics (MD) simulations were conducted to examine Li^+^ diffusion and hydration behavior at temperatures corresponding to different solar intensities. As illustrated in Figure 5h, both structural and kinetic rearrangements of water molecules evolve over time, leading to progressively more ordered hydration structures surrounding Li^+^ ions. With increasing temperature, the interaction between Li^+^ ions and neighboring water molecules becomes stronger, resulting in the formation of more compact and stable hydration shells (Figure 5c). This behavior is further supported by radial distribution function (RDF) analysis (Figure 5d), which reveals an increased population of water molecules within the first and second hydration shells at elevated temperature, consistent with enhanced inner‐sphere coordination.

Meanwhile, interfacial water evaporation is thermally accelerated, as shown in Figure 5g, with increased thermal energy enhancing the mobility of surface water molecules and lowering the interfacial tension. However, experimental data indicate that although evaporation is initially enhanced under photothermal conditions, it gradually declines over time under excessive solar intensity. Upon evaporation, Li^+^ ions become locally concentrated, leading to enhanced hydration (Figure 5e). To gain deeper insight into how Li^+^ ion concentration affects hydration behavior and lithium adsorption experiments, surface tension measurements were conducted in Figure 5f. As the Li^+^ concentration increased from 0 to 1000 mg/L, the surface tension of the solution progressively decreased. This trend suggests that the presence of metal ions disrupts hydrogen bonding among water molecules and reduces intermolecular cohesion at the liquid–air interface, thereby facilitating water molecule escape and accelerating evaporation [24]. Under photothermal conditions, high salt concentrations enhance interfacial evaporation by lowering surface tension, ultimately improving water removal efficiency. Notably, as demonstrated earlier in Figure 3b, Mo‐LDH@Sponge also exhibits improved lithium adsorption at higher Li^+^ concentrations. The concurrent enhancement of evaporation and adsorption performance underscores a synergistic interplay between the material's photothermal and ion‐binding functionalities, making it particularly promising for integrated solar‐driven evaporation and lithium recovery applications.

To summarize, this behavior arises from two competing mechanisms: (i) evaporation‐driven salt concentration increases the local Li^+^ density, which stabilizes additional water molecules within the hydration shells; and (ii) progressive depletion of free water reduces the amount of water available for continued evaporation. Consequently, the system exhibits a two‐stage dynamic response – an initial thermally driven enhancement of evaporation, followed by a concentration‐induced stabilization of hydration structures that suppress further evaporation. These insights highlight the delicate interplay between water availability and lithium mobility under variable solar input and provide a mechanistic understanding of the material's dual‐function operation under solar irradiation.

**FIGURE 6:**
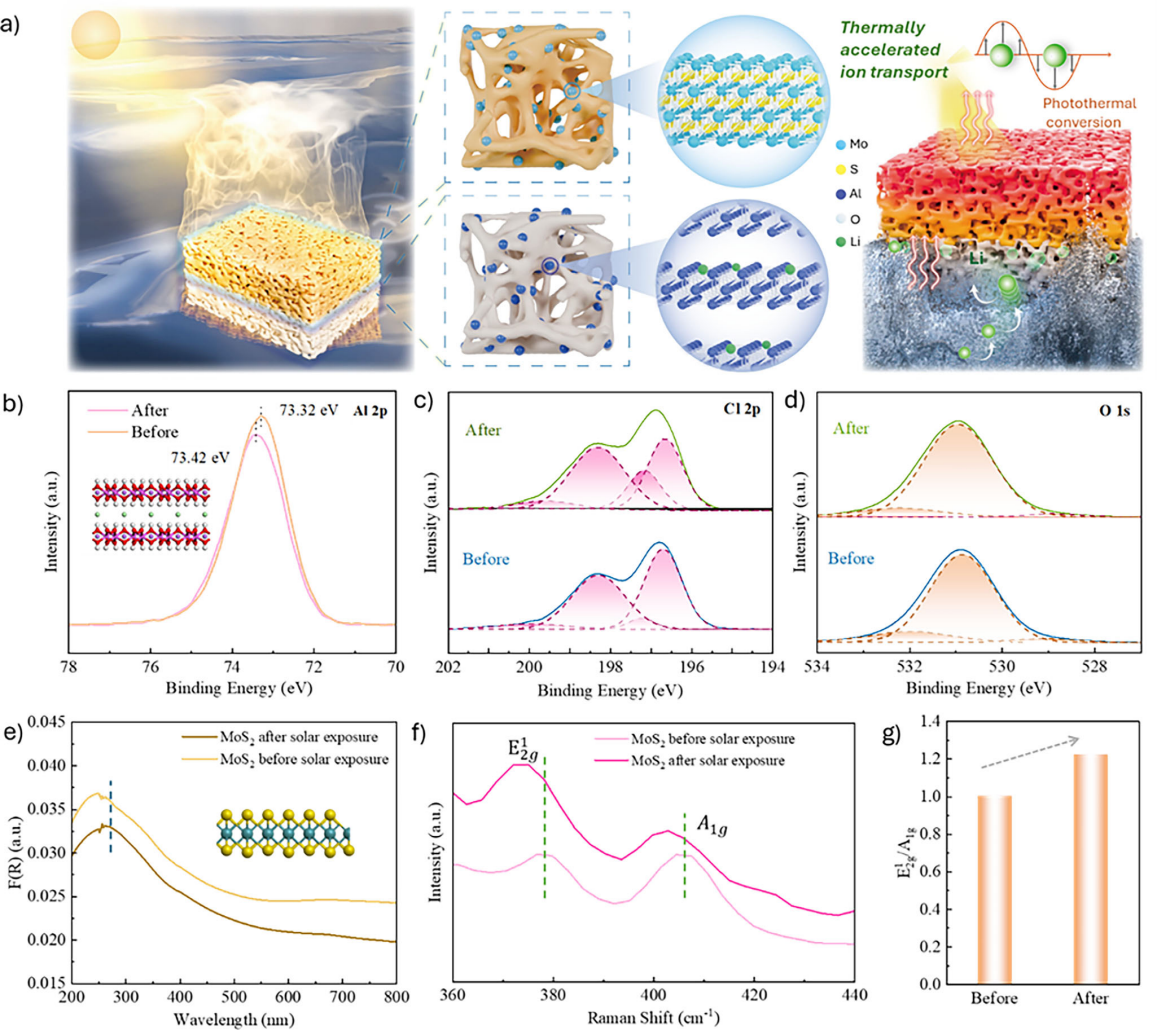
(a) Schematic illustration of the lithium adsorption mechanism enhanced by solar evaporation in the dual‐functional Mo‐LDH@Sponge; (b‐d) High‐resolution XPS spectra of Al 2p, Cl 2p, and O 1s for Li/Al‐LDH before and after lithium adsorption; (e) UV‐Vis‐NIR spectra, (f) Raman spectra, and (g) $E_{2g}^{1}/A_{1g}$ intensity ratio variation of MoS_2_ before and after solar exposure.

Figure 6a illustrates the working mechanism of the dual‐functional Mo‐LDH@Sponge, which integrates solar‐driven evaporation with selective lithium adsorption through a vertically layered architecture. The upper MoS_2_@Sponge layer serves as an efficient photothermal converter, harvesting solar energy to generate localized heat that drives water evaporation and enables desalination. Simultaneously, the underlying LDH@Sponge selectively captures Li^+^ ions through Al–O vacancies. The generated photothermal heat not only enhances the interfacial evaporation rate but also modulates the physicochemical environment of the solution. Specifically, photothermal heating increases the water molecules mobility and reduces interfacial tension, thereby accelerating water evaporation and concentrating Li^+^ ions in the residual solution. At later stages, this restructuring leads to increased solution viscosity and reduced ionic diffusivity. Consequently, the system exhibits a dynamic balance: at the initial stage, thermal energy simultaneously promotes Li^+^ transport and water evaporation; however, under higher irradiation intensities, rapid solvent loss and increasingly stabilized hydration shells gradually constrain further adsorption under excessive solar intensity (e.g., 1 sun). This dual‐stage behavior underpins a light‐regulated “photothermal‐concentration‐adsorption” mechanism, in which solar energy not only drives water evaporation but also governs ion mobility and local hydration structures. Through this vertically integrated Mo‐LDH@Sponge design, the Mo‐LDH@Sponge system effectively exploits photothermal–adsorptive coupling to achieve high‐efficiency desalination and lithium recovery under variable solar input.

To elucidate the chemical‐state evolution and synergistic functionality of Mo‐LDH@Sponge following solar light exposure and lithium adsorption, the material was systematically characterized before and after the reaction. As shown in Figure 6b–d, high‐resolution XPS spectra reveal distinct changes in the Al 2p, Cl 2p, and O 1s regions of the Li/Al‐LDH component. After lithium adsorption, a slight shift in the Al 2p peak indicates modulation of the local electronic environment of Al species, suggesting an interaction between Li^+^ ions and the Al–O coordination framework of the LDH layers [25]. Quantitative XPS atomic percentages before and after adsorption (Table S5) show that the relative contents of Al and O remain essentially unchanged, confirming that the observed spectral shifts arise from localized electronic restructuring rather than bulk compositional variation. Meanwhile, changes in the Cl 2p spectral features are observed, which are attributed to redistribution or coordination variation of interlayer chloride species originating from the chloride‐based simulated brine, rather than bulk compositional changes. The O 1s spectrum exhibits increased intensity and peak broadening after adsorption, reflecting alterations in hydroxyl and lattice oxygen states associated with Li^+^ interaction and local coordination rearrangement. Collectively, these spectroscopic features support that lithium uptake occurs predominantly within the LDH layers and is accompanied by localized electronic restructuring, while the overall framework of the material remains structurally intact [26].

Figure 6e shows the UV–vis–NIR spectra of MoS_2_ before and after solar exposure. A noticeable decrease in reflectance across the UV‐visible region (particularly from 250 to 400 nm) and a downward shift of the F(R) curve indicate enhanced light absorption after irradiation. This enhancement is likely attributed to light‐induced surface modifications or an increase in defect states, which promote electron‐hole generation and transfer, thereby improving photothermal conversion efficiency and photo responsiveness under operational conditions [27]. In addition, Raman spectra result shown in Figure 6f,g further reveals structural modifications in MoS_2_. After illumination, the intensities of both $E_{2g}^{1}$ and *A*_1*g*_ vibrational modes increase, with a more pronounced enhancement in the $E_{2g}^{1}$ peak, resulting in a higher $E_{2g}^{1}/A_{1g}$ intensity ratio. This suggests an increase in lattice ordering and a reduction in interlayer coupling, potentially due to water desorption or relaxation of lattice stress. The enhanced in‐plane vibrational response also reflects improved energy transfer capability under photothermal excitation [28, 29]. Together, these spectral changes corroborate the improved structural stability and photothermal performance of MoS_2_, reinforcing its suitability for sustained operation in solar‐driven enhanced lithium adsorption systems.

## 3 Conclusion and Future Perspective

This study demonstrates a Mo‐LDH@Sponge composite that integrates photothermal interfacial evaporation with lithium adsorption for selective lithium recovery from saline water. The main conclusions can be summarized as follows:

- Material design and functional integration: A hierarchically structured Mo‐LDH@Sponge was developed by vertically assembling a flower‐like MoS_2_ photothermal layer with a Li/Al‐LDH adsorption layer on a porous sponge substrate. This integrated architecture enables simultaneous solar‐driven water evaporation and lithium capture within a single platform, providing an efficient and compact configuration for solar‐assisted lithium recovery.
- Adsorption behavior regulated by photothermal hydration effects: The lithium capture process is governed by a hydration‐controlled adsorption mechanism induced by photothermal interfacial heating. Under moderate illumination, photothermally enhanced evaporation promotes Li^+^ transport toward the adsorption layer, leading to improved uptake. In contrast, excessive heating strengthens ion‐solvent coordination and reduces Li^+^ mobility, resulting in diffusion‐limited adsorption. Molecular dynamics simulations combined with experimental results reveal that thermally driven changes in the hydration environment play a decisive role in regulating adsorption kinetics and selectivity.
- Selectivity, stability, and practical relevance: The Mo‐LDH@Sponge composite exhibits pronounced selectivity toward lithium over divalent cations, which is primarily associated with the surface chemistry and size‐exclusion characteristics of the LDH lamellae. The material also maintains structural stability and reusability during repeated operation, indicating its potential for solar‐powered lithium extraction. These findings illustrate how coupling interfacial photothermal evaporation with hydration‐tuned adsorption offers a viable design strategy for energy–water–resource coupling systems aimed at recovering lithium and other critical elements from complex aqueous environments.
- Scope and future directions: The present study focuses on fundamental integration and mechanistic understanding under controlled conditions. Long‐term cycling durability and performance evaluation in realistic brine systems are beyond the scope of this work and are currently being explored in ongoing parallel studies.

## 4 Experimental Section

### 4.1 Design and Synthesis of Mo‐LDH@Sponge Composite

MoS_2_ was synthesized via a hydrothermal method. 2 mmol of (NH_4_)_6_Mo_7_O_24_·4H_2_O and 60 mmol of CH_4_N_2_S were dissolved in 72 mL of deionized water (DIW) under vigorous stirring. The solution was then transferred to a 100 mL Teflon‐lined stainless‐steel autoclave and heated at 200°C for 24 h. After cooling to room temperature, the black precipitate was collected by centrifugation, washed several times with DIW and ethanol, and vacuum‐dried to yield MoS_2_ powder. Li/Al‐LDH was prepared via a one‐step co‐precipitation method. A mixed aqueous solution of Li^+^ and Al^3+^ salts (Li/Al molar ratio = 0.24) was slowly added dropwise to a concentrated NaOH solution under continuous stirring at ∼45°C. The reaction was terminated at pH ≈ 5, followed by an aging and washing process. The resulting slurry was filtered and dried to obtain the Li/Al‐LDH powder. Commercial cellulose sponges were rinsed with DIW and soaked in ethanol to remove surface‐bound organic residues, then dried at 80°C for 12 h. This cleaning‐drying cycle was repeated three times to ensure complete removal of contaminants. Subsequently, MoS_2_ and Li/Al‐LDH powders were dispersed in DIW via ultrasonication to form uniform suspensions. The pretreated sponges were sequentially immersed in the suspensions and subjected to ∼50 compression‐release cycles to promote deep infiltration and firm adhesion of the active materials. The modified sponges were dried at 60°C to yield MoS_2_@Sponge and LDH@Sponge, respectively. Finally, the two functionalized components were bonded together using a water‐resistant adhesive to fabricate the bilayer composite, referred to as Mo‐LDH@Sponge.

### 4.2 Adsorption Experiment Setups and Analysis

Solar evaporation and lithium adsorption experiments were conducted using a custom‐fabricated transparent acrylic chamber. The Mo‐LDH@Sponge composite was irradiated in a dark space using a solar simulator (Newport 3M, ORIEL Sol3A, Xe lamp) equipped with an AM 1.5G filter, with the output power controlled by a power meter. The simulator was operated at a fixed working distance recommended by the manufacturer, providing a uniform illumination area of approximately 100 cm^2^ at the sample plane. Under these conditions, an input electrical power of 600 W corresponds to an irradiance of approximately 1 sun (≈100 mW/cm^2^) at the sample surface, consistent with the nominal operating specifications of the Sol3A system. Samples were placed in Petri dishes containing simulated saline solutions, alongside a blank control without the composite. To investigate the light‐intensity dependence, additional experiments were conducted at reduced lamp powers of 200 (0.33 sun), 400 (0.67 sun), and 600 W (1 sun), corresponding to sub‐sun to one‐sun irradiation conditions. The steady‐state solar‐to‐vapor conversion efficiency η was calculated as the ratio of the heat consumed for water evaporation (including both latent and sensible heat contributions) to the incident solar power [30]:

(2) $$\eta =\frac{m\cdot h_{Iv}}{I}$$

where *m* is the steady‐state mass flux of evaporated water (kg/m^2^·s), *I* is the incident solar irradiance (W/m^2^), and *h_lv_* is the effective evaporation enthalpy (J/kg), which includes both latent heat and sensible heat [31]:

(3) $$h_{Iv}=\lambda \left(T_{s}\right)+C_{p}\cdot \left(T_{s}-\;T_{0}\right)$$

here, λ(*T_s_*) is the temperature‐dependent latent heat of vaporization of water at the steady‐state surface temperature *T_s_*, *C_p_* is the specific heat capacity of water (*C_p_* =  4180 J/kg·K), and *T*_0_ is the initial surface temperature (at *t*  =  0). The latent heat term was estimated using the commonly adopted empirical correlation:

(4)

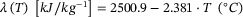




Lithium concentrations in the feed solution were varied at 500, 700, and 1000 mg/L to evaluate concentration effects. During testing, the lower LDH@Sponge of the composite remained fully immersed due to its high hydrophilicity, initiating the lithium adsorption process. Kinetic experiments were conducted by collecting aliquots at predetermined time intervals. The Li^+^ concentrations were quantified, and the lithium adsorption capacity (mg/dm^3^) was calculated based on the difference from the initial concentration using Equation (5):

(5) $$q=\frac{v_{0}\cdot C_{0}-v_{t}\cdot C_{t}}{S}\;$$

where *C*_0_ and *C_t_* (mg/L) refer to the Li^+^ ion concentration in the initial solution and the solution at time *t*, respectively. *v*_0_ and *v_t_* (L) is the volume of the solution in the chamber at the initial time and time *t*, and *S* (dm^3^) is the volume of Mo‐LDH@Sponge. To assess the reusability of the Mo‐LDH@Sponge composite, lithium desorption experiments were carried out using eluents containing low concentrations of Li^+^. After adsorption, the material was immersed in the desorption solution, and the amount of lithium released was quantified according to Equation (S1).

To evaluate the lithium selectivity of the Mo‐LDH@Sponge composite, adsorption experiments were performed in mixed salt solutions containing K^+^, Na^+^, Ca^2+^, and Mg^2+^ at concentrations equal to that of Li^+^. The composite was exposed to the multicomponent solution, and aliquots were collected at designated time intervals for ion concentration analysis. The adsorption performance toward different cations was compared, and lithium selectivity was quantitatively assessed using separation factors *K_Me_* (Me = Li, Na, K, Ca, Mg) and selective adsorption coefficients $a_{Me}^{Li}$, as defined in Equations (S2)and (S3), respectively.

To visualize the positive contribution of solar‐driven evaporation to Li^+^ ion uptake during the desalination process, we define the Evaporation‐Assisted Li^+^ Adsorption Gain (EAG), expressed as mg Li^+^ per g H_2_O reacted in per dm^3^ Mo‐LDH@Sponge. As shown in Equation (S3). EAG quantifies the additional lithium adsorption attributed to photothermal evaporation, normalized by the amount of water lost due to evaporation:

(6) $$EAG=\frac{q_{eavp}-q_{dark}}{m_{evap}}$$

where, ​ *q_evap_* and *q_dark_* (mg/dm^3^) represent the lithium adsorption capacities of the illuminated (evaporation) and dark (non‐evaporation) groups, respectively, while *m_evap_* (g) denotes the total mass of evaporated water or the product of evaporation rate and exposed surface area. A higher EAG value indicates a greater lithium adsorption gain per unit of evaporated water, reflecting the efficiency of photothermal‐driven enrichment during solar irradiation.

### 4.3 Solar‐Driven Evaporation Experiments

Under solar simulator irradiation, lithium adsorption occurred concurrently with photothermal‐driven water evaporation, enabling simultaneous desalination. To investigate the system's performance under varying conditions, a series of experiments were conducted using different solar intensities (0.33, 0.67, and 1.00 sun) and salt concentrations (500, 700, and 1000 mg/L). Real‐time monitoring of mass loss due to water evaporation provided direct insight into the desalination and photothermal efficiency. The extent of water evaporation and desalination performance was evaluated using two parameters: mass change (%) and evaporation rate (g/dm^3^·h), which were calculated based on Equations (S4) and (S5), respectively. These metrics offer a quantitative measure of the system's evaporative water loss and its capacity to drive desalination under solar‐driven conditions.

### 4.4 Spectroscopic and Microscopic Characterization Techniques

The microscopic morphology of the Mo‐LDH@Sponge composite was characterized using scanning electron microscopy (SEM, JEOL, Peabody, MA, USA), operated at accelerating voltages ranging from 3.0 to 20.0 kV, to observe surface topography and pore structures. Transmission electron microscopy (TEM, JEOL JEM‐2100, USA) equipped with high‐angle annular dark‐field (HAADF) imaging was employed to examine internal microstructures and crystalline domains. High‐resolution TEM (HRTEM), selected area electron diffraction (SAED), and TEM‐based energy‐dispersive X‐ray spectroscopy (TEM‐EDS mapping) were further used to resolve atomic lattice fringes, assess crystallinity, and visualize the nanoscale spatial distribution of elements.

Dynamic contact angle measurements were performed using a Theta Flow Tensiometer to evaluate the surface wettability under fluid‐solid interaction. The surface tension of liquid samples was measured via the pendant drop method. Infrared thermal imaging (HIKMICRO B10) was employed to monitor surface temperature distributions under simulated solar irradiation, enabling visualization of photothermal conversion behavior over time. UV–vis–NIR spectroscopy (Agilent Cary 5000) was conducted to assess light absorption properties over a broad spectral range. Raman spectroscopy (XploRA PLUS Raman microscope) was employed to analyze molecular vibrations and phase composition, while X‐ray photoelectron spectroscopy (XPS, PHI Quantera Hybrid system) provided insights into surface chemical states and elemental bonding environments. Ion concentrations in solutions, including lithium, were quantified using inductively coupled plasma mass spectrometry (ICP‐MS, Agilent 7900). All characterization measurements were conducted in triplicate or more to ensure data reliability and experimental reproducibility.

### 4.5 Modeling and Simulation of Temperature‐Adsorption Coupling Behavior

Molecular dynamics simulations (MDs) were performed to investigate the transport behavior of lithium ions and the structural dynamics of water molecules under different temperatures induced by solar irradiation, as well as the effect of varying lithium concentrations. The simulation system was constructed using 500 water molecules (SPC model) and a series of lithium ions, built within an amorphous cell. The COMPASS force field was employed to accurately describe the conformational and vibrational behavior of atoms. The simulations were performed in an NVT ensemble, maintaining a constant number of particles, volume, and temperature to avoid trajectory distortion. Temperature control was achieved using the Nose‐Hoover thermostat, and the initial atomic velocities followed the Maxwell‐Boltzmann distribution. A time step of 0.001 ps was adopted, with periodic boundary conditions applied along the x, y, and z axes. Non‐bonded interactions were modeled using a van der Waals cutoff distance of 12.5 Å, while electrostatic interactions were treated via the atom‐based method and the Ewald summation technique. To explore the influence of thermal conditions and ionic strength, simulations were conducted at four different temperatures (298, 308, 318, and 328 K) and five Li^+^ ion concentrations (1, 2, 3, 4, and 10 ions.

### 4.6 Statistical Analysis

All experiments were conducted in triplicate unless otherwise stated. Data are reported as mean values. Graphical data processing and plotting were performed using Origin software (OriginLab). No additional statistical tests were applied, as the study focuses on mechanistic trends rather than hypothesis testing.

## Author Contributions

Y.P. conceived the overall research idea, designed and carried out the majority of the experiments, analyzed the data, and led the writing and organization of the manuscript. Y.Z. assisted with part of the experimental work and data collection. G.L. provided key experimental facilities and technical support, and contributed to manuscript revision. W.Z. performed the theoretical calculations, contributed to data interpretation, and participated in writing and revising the manuscript. W.Z. supervised the project, secured funding and experimental resources, provided critical guidance throughout the study, and revised the manuscript with constructive suggestions.

## Conflicts of Interest

The authors declare the following financial interests/personal relationships which may be considered as potential conflicts of interest: Wencai Zhang is a paid consultant for and has an equity interest in Austin Elements Inc., a research partner on this award (DE‐FE0032492).

## Supporting information




**Supporting File 1**: advs74190‐sup‐0001‐SuppMat.docx

**Supporting File 2**: advs74190‐sup‐0002‐VideoS1.mp4

## Data Availability

The data that support the findings of this study are available from the corresponding author upon reasonable request.
