# Supplementary material for: Selective Lithium Recovery via Photothermal Evaporation and Hydration‐Controlled Adsorption
Source: Adv Sci (Weinh). 2026 Feb 10;13(20):e20066. doi: 10.1002/advs.202520066 (PMC13067783; doi:10.1002/advs.202520066)
Supplement: Supplementary file 1 — Supporting File 1: advs74190‐sup‐0001‐SuppMat.docx [file ADVS-13-e20066-s001.docx]

**Supporting Information**

**Selective Lithium Recovery via Photothermal Evaporation and Hydration-Controlled Adsorption**

^1^Yanan Pan, ^2^Yue Zhang, ^2, 3, 4, 5^ Guoliang Liu, ^1,^ *Weiquan Zhan, ^1,^ *Wencai Zhang

*^1^ Department of Mining and Minerals Engineering, Virginia Polytechnic Institute and State University, Blacksburg, Virginia 24061, United States*

*^2^ Department of Chemistry, Virginia Polytechnic Institute and State University, Blacksburg, Virginia 24061, United States*

*^3^ Department of Chemical Engineering, Virginia Polytechnic Institute and State University, Blacksburg, Virginia 24061, United States*

*^4^ Department of Materials Science and Engineering, Virginia Polytechnic Institute and State University, Blacksburg, Virginia 24061, United States*

*^5^ Macromolecules Innovation Institute, Virginia Polytechnic Institute and State University, Blacksburg, Virginia 24061, United States*

*Corresponding authors:

W. Zhan; E-mail: zhan_weiquan@163.com

W. Zhang; E-mail: wencaizhang@vt.edu

1. Experimental Results

**Video**: Simulated 3D process for dual function of desalination and lithium capture (see attached file).


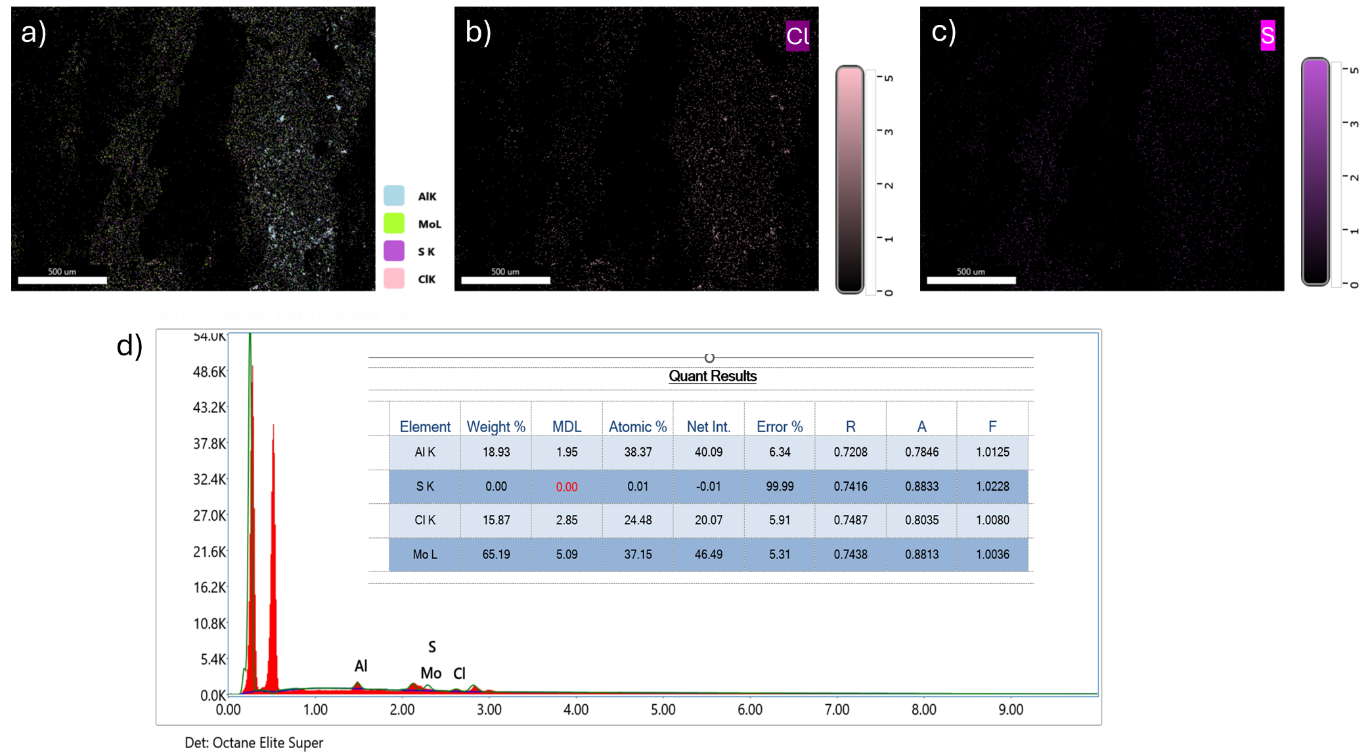


**Figure S1.** (a-c) SEM-EDS mapping of Mo-LDH@Sponge materials; (d) EDS spectra and elemental composition of Mo-LDH@Sponge materials.


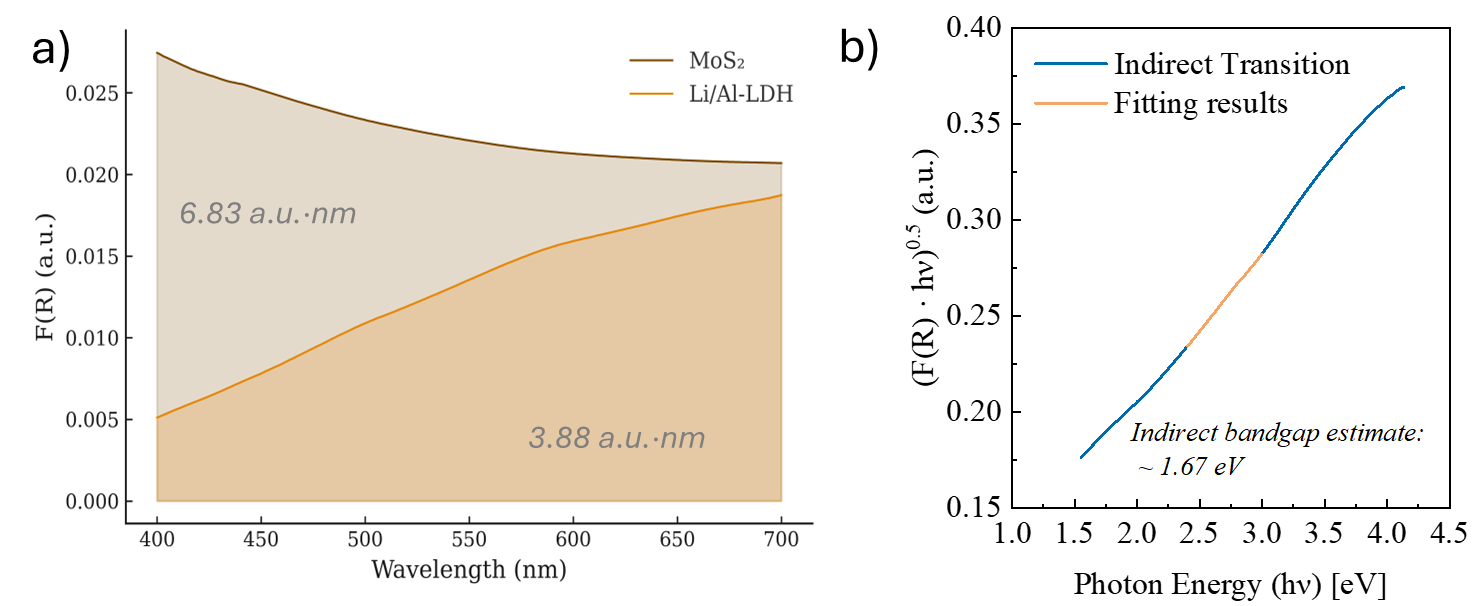


**Figure S2.** (a) UV–Vis diffuse reflectance spectra of MoS_2_ and Li/Al-LDH; (b) Tauc plot of MoS_2_ based on the Kubelka–Munk function.


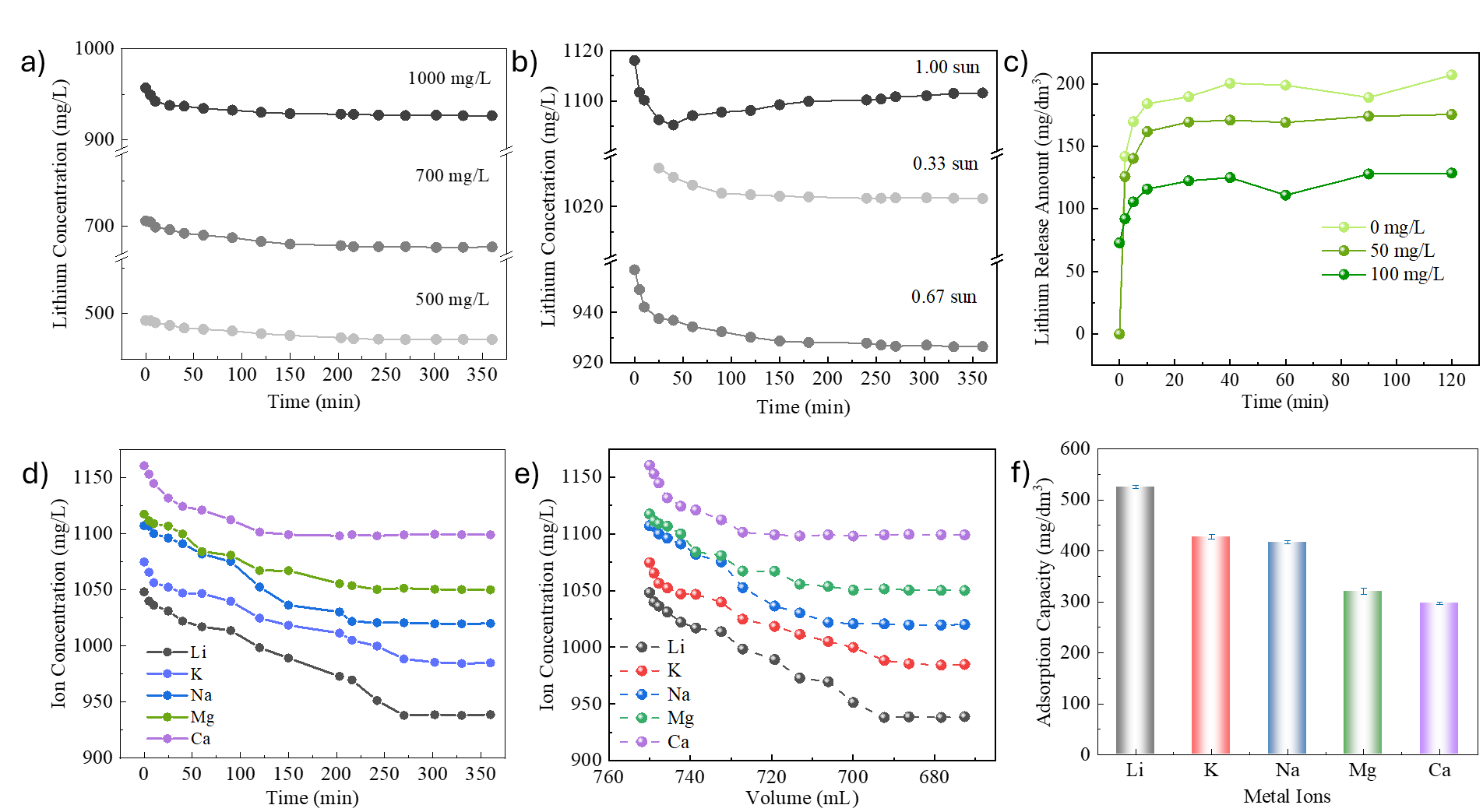


**Figure S3.** (a) Time-dependent lithium concentration profiles under varying initial lithium concentrations; (b) Lithium concentration changes over time under different solar illumination intensities. (c) Lithium release/desorption amount as a function of time at different initial lithium concentrations in the desorption solution; (d) Changes in different ion concentrations over time; (e) Ion concentration variation as a function of remaining solution volume; (f) Comparison of adsorption capacities for different metal ions.


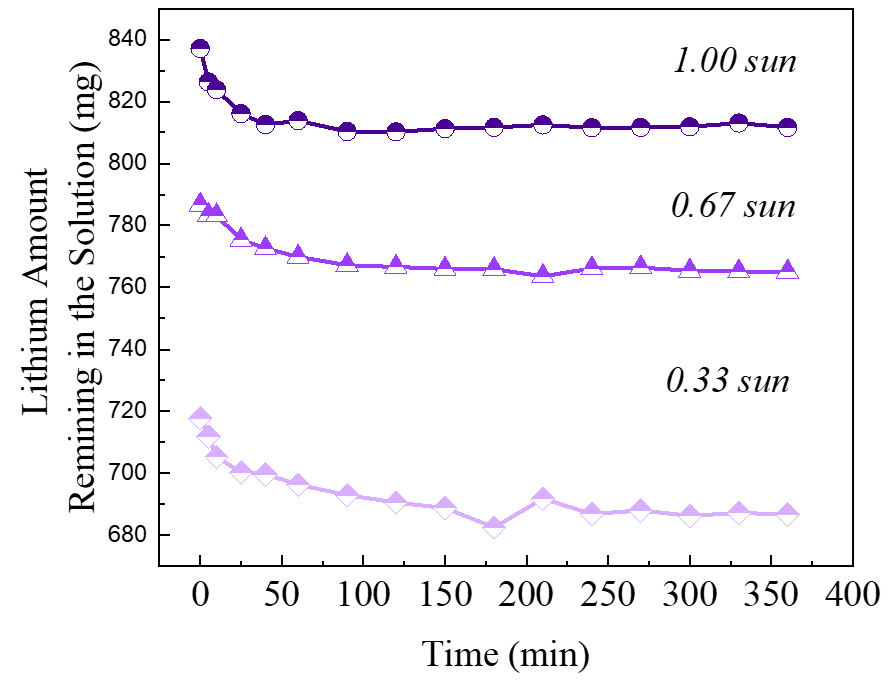


**Figure S4.** Lithium amount remaining in the solution at different intensities.

**Table S1.** Parameters for Weber-Morris intraparticle diffusion model fitting of lithium adsorption under various solar intensities.

| Solar intensities | k1 (mg/(dm^3^🞗min^1/2^)) | k2 (mg/(dm^3^🞗min^1/2^)) | k3 (mg/(dm^3^🞗min^1/2^)) |
| --- | --- | --- | --- |
| 0.33 sun | 15.02 | 10.96 | 1.19 |
| 0.67 sun | 19.64 | 13.69 | 3.79 |
| 1.00 sun | 24.92 | 4.77 | -1.37 |

1. Equations

The desorption amount $d$ (mg/dm^3^) was calculated according to $Eq. (S1)$:

$d=\frac{v{\cdot(C}_{de}-C_{d0})}{S}$ (S1)

where $C_{de}$ (mg/L) refers to the ion concentration in the final solution, $C_{do}$ (mg/L) refers to the initial lithium concentration in the desorption feed solution, $S$ (dm^3^) is volume of the solution, $v$ (L) is the volume of desorption feed solution.

$K_{Me}=\frac{(C_{0}-C_{e})\cdot v}{C_{e}\cdot m}$ (S2)

$a_{Li}^{Me} =\frac{K_{Li}}{K_{Me}}$ (S3)

where $C_{0}$ (mg/L) and $C_{e}$ (mg/L) refer to then initial ion concentration and the equilibrium concentration, $m$ (g) is the added adsorbents mass, $v$(mL) is the solution volume.

$Mass Change \left( \% \right)=100\times\frac{m_{0}-m_{t}}{m_{0}}$ (S4)

$Evaporation Rate \left( \% \right)=\frac{\Delta m}{V\times t}$ (S5)

where $m_{0}$ and $m_{t}$​ represent the initial and remaining mass of the solution at time $t$, respectively (g); $\Delta m$ is the net mass of evaporated water (g); $V$ is the material volume of the material (dm^3^); and $t$ is the evaporation time (h).

1. Raw data for steady-state evaporation calculation

**Table S2.** Time-dependent mass change of water during photothermal evaporation of Mo-LDH@Sponge under different solar intensities.

| Time/min | Mass Change/g | Mass Change/g | Mass Change/g |
| --- | --- | --- | --- |
|  | 0.33 sun | 0.67 sun | 1.00 sun |
| 0 | 0 | 0 | 0 |
| 5 | 2.71 | 0.04 | 1.47 |
| 10 | 3.09 | 1.81 | 3.11 |
| 25 | 4.58 | 5.68 | 6.97 |
| 40 | 6.04 | 9.65 | 13.15 |
| 60 | 8 | 15.54 | 21.03 |
| 90 | 10.28 | 24.08 | 34.06 |
| 120 | 12.7 | 33.45 | 47.64 |
| 150 | 15.33 | 43.56 | 61.76 |
| 180 | 17.84 | 61.35 | 76.3 |
| 240 | 22.66 | 66.51 | 90.17 |
| 255 | 24.34 | 75.33 | 105.09 |
| 270 | 25.86 | 84.1 | 120.31 |
| 302 | 28.66 | 95.37 | 135.38 |
| 330 | 31.6 | 104.75 | 149.67 |
| 360 | 34.43 | 114.71 | 165.25 |

**Table S3.** Time-dependent surface temperature evolution of Mo-LDH@Sponge under different solar intensities.

| Time/min | Surface Temperature/℃ | Surface Temperature/℃ | Surface Temperature/℃ |
| --- | --- | --- | --- |
|  | 0.33 sun | 0.67 sun | 1.00 sun |
| 0 | 21.3 | 25.5 | 22.7 |
| 5 | 33.8 | 44.1 | 50 |
| 10 | 36.3 | 46.7 | 52.9 |
| 15 | 38.6 | 48.5 | 54.7 |
| 20 | 39.3 | 48.9 | 54.2 |
| 25 | 39.8 | 50.4 | 55.5 |
| 30 | 40.8 | 49.6 | 56.8 |
| 35 | 41.2 | 51.1 | 56.5 |
| 40 | 41.1 | 50 | 56.2 |
| 45 | 40.9 | 49.6 | 56.4 |
| 50 | 41.4 | 50.3 | 56.7 |
| 55 | 41.9 | 49.9 | 56.5 |
| 60 | 41 | 50.4 | 56.7 |

For the incident irradiance, 1 sun corresponds to 100 mW/m^2^ (1000 W/m^2^). Accordingly, the irradiance levels of 0.33 sun, 0.67 sun, and 1.00 sun correspond to 330, 670, and 1000 W/m², respectively. The steady-state evaporation regime was defined as the time window from 240 to 360 min, during which the mass loss exhibited a linear dependence on time. Linear fitting of the mass loss data yielded steady-state mass loss rates of k=0.0971, 0.3906, and 0.5985 g/min under 0.33 sun, 0.67 sun, and 1.00 sun irradiation, respectively.

The steady-state mass flux $m$ (kg/m^2^·s) was calculated as:

$m=k/(1000 \cdot60 \cdot A)$ (S6)

where $A$represents the normalization area.

Based on the measured surface temperatures, the effective evaporation enthalpy ($h_{lv}$) values, including both latent and sensible heat contributions, were calculated as:

$h_{Iv}$ (0.33 sun) = 2.486 × 10⁶ J/kg

$h_{Iv}$ (0.67 sun) = 2.485 × 10⁶ J/kg

$h_{Iv}$ (1.00 sun) = 2.508 × 10⁶ J/kg

The projected illuminated area of the device ($A_{proj}$) was 0.01 m². Due to the 3D porous structure, an effective evaporation area $A_{eff}$ was introduced to ensure energy self-consistency. The effective area was determined as:

$A_{eff}=A_{proj} \cdot\eta_{proj}(1.00 sun)$= 0.0250 m^2^

Accordingly, the corrected steady-state solar-to-vapor conversion efficiencies were obtained as:

$\eta$(0.33 sun) = 48.7%

$\eta$ (0.67 sun) = 96.5%

$\eta$ (1.00 sun) = 100.0%

1. Comparison of photothermal-assisted lithium adsorption materials reported in the literature.

**Table S4.** Comparison of photothermal-assisted lithium adsorption materials reported in the literature.

| ***Ref.*** | **Material system** | **Photothermal component** | **Adsorption mechanism** | **Li adsorption capacity** | **Selectivity / key feature** | **Advantages / limitations** |
| --- | --- | --- | --- | --- | --- | --- |
| Song et.al.^1^ | STLES (Solar transpiration–powered lithium extraction and storage) | Al nanoparticle–decorated anodic aluminum oxide (AAO) transpiration evaporator | Pressure-driven extraction (not direct adsorption) | ~33.2 mg Li m⁻² day⁻¹ (from ~3 mg/L brine); water flux up to ~1.8 L m⁻² h⁻¹ | High Li⁺ selectivity governed by nanofiltration membrane energy barrier | Passive solar operation, high scalability and stability (>500 h); relies on membrane selectivity rather than intrinsic Li-specific adsorption |
| Xia et.al.^2^ | Solar microevaporator–integrated Li⁺ sieve (HTO@NMC-PS) | N-doped mesoporous carbon (NMC) photothermal layer | Temperature-enhanced Li⁺ ion exchange on lithium-ion sieve under solar evaporation | ~28.7 mg/g (1 sun); >8 mg/g from natural salt-lake brine | High Li⁺ selectivity from ion-sieve chemistry; photothermal nanoconfinement | Solar-enhanced kinetics and water recycling; higher system complexity than standalone adsorbents |
| Chen et. al.^3^ | Solar-driven direct extraction felt (DEF) | Dark-colored Li₁.₆Mn₁.₆O₄ (LMO)–coated floating fibrous felt | Temperature-enhanced Li⁺ ion exchange on lithium-ion sieve assisted by solar evaporation | Up to ~520 mg/m² under 2 sun (indoor); effective extraction from low-Li simulated seawater outdoors | High Li⁺ selectivity from LMO ion-sieve chemistry; reduced concentration polarization | Pump-free, floating and scalable; performance degradation associated with Mn loss during cycling |
| Zhang te.al.^4^ | Solar-driven membrane-based Li extraction evaporator (PANI/PA membrane system) | Vertically aligned polyaniline (PANI) nanoarray photothermal layer | Membrane-based Li⁺/Mg²⁺ separation coupled with solar-driven evaporation (not direct adsorption) | LiCl crystallization rate up to ~186 g m⁻² h⁻¹ (3 sun) | High Li⁺ selectivity from polyamide nanofiltration membrane (size sieving + Donnan exclusion) | Direct LiCl recovery from high-salinity brine; relies on membrane selectivity and multicomponent system complexity |
| Zhao et.al. ^5^ | Solar-driven LIG/MnO₂ lithium extraction device | Laser-induced graphene (LIG) with MnO₂ ion-sieve nanoparticles | Solar-enhanced Li⁺ ion exchange on Mn-based ion sieve coupled with interfacial evaporation | ~13.48 mg/g (artificial brine, 1 sun) | High Li⁺ selectivity from MnO₂ ion-sieve chemistry; photothermal concentration at interface | High evaporation rate and good cycling stability; relies on Mn-based ion sieve and multicomponent 3D device structure |
| Zhou et.al. ^6^ | Solar-driven pump integrated photothermal evaporator | Photothermal evaporator layer: carbon nanotubes (CNTs) and lithium ion-sieves (H_4_Ti_5_O_12_) | Evaporation-induced local Li⁺ enrichment and simultaneous adsorption | 50.1 ± 1.6 mg/g within 5 h under 1 sun | Good adsorption selectivity and operational stability during coupled evaporation–adsorption | Integrated freshwater production and Li recovery |
| Li et.al. ^7^ | Photothermal ion pumps (PIPs) for seawater Li extraction | Hydrophobic PVDF/Fe₃O₄ photothermal nanofibrous shell | Solar-enhanced Li⁺ ion-sieve trapping (HMO or HTiO) coupled with evaporation-driven enrichment | ~24.0 ± 1.1 mg/g (HMO-based PIPs, seawater-relevant conditions) | Ultrahigh Li⁺ selectivity (Li⁺/Mg²⁺ ≈ 1.3 × 10⁴); mitigated concentration polarization via gravity–evaporation synergy | Excellent durability and low energy consumption; device architecture relatively complex and relies on ion-sieve chemistry |
| Wang et.al. ^8^ | Photothermal-enhanced electrochemical lithium extraction system (ELiES) | Polypyrrole (PPy)–coated FePO₄ electrode | Solar-assisted electrochemical Li⁺ intercalation (not direct adsorption) | 5.84 mmol/g (~40.5 mg/g) under 1 sun at 5 °C; 3.30 mmol/g at Mg/Li = 100 | High Li⁺/Mg²⁺ selectivity (~124) enabled by PPy-regulated ion transport and reduced polarization | Excellent low-temperature performance and long-term durability (81% retention after 5000 cycles); requires electrochemical cell and external bias |
| Zhong et.al. ^9^ | Photothermal sandwich sieve device (LIG/HKUST-1) | Laser-induced graphene (LIG) photothermal layer | Size-selective permeation through sub-nano HKUST-1 MOF channels coupled with evaporation-driven Li⁺ enrichment (not bulk adsorption) | Up to ~1467 mg/m² per cycle (1 sun); ~1698 mg/m² under 3 sun | High Li⁺/Mg²⁺ selectivity from MOF window size matching hydrated Li⁺ | Integrated solar utilization and good cycling stability; performance relies on membrane integrity and multicomponent device architecture |
| Yu et.al. ^10^ | Adsorption-responsive photothermal ion pump (APIP) | Hydrogel-based photothermal absorber with confined HMO | Solar-enhanced Li⁺ ion-sieve adsorption coupled with evaporation-induced enrichment and swelling-assisted site exposure | ~34 mg/g (based on HMO, real seawater); up to ~4.14 mmol/g in simulated seawater | High Li⁺ selectivity from HMO ion-sieve chemistry; adsorption-responsive swelling and suppressed Mn dissolution | Reversible extraction, fast kinetics, excellent cycling stability; relies on Mn-based ion sieves and multicomponent hydrogel architecture |
| Xing et.al. ^11^ | 3D solar evaporator with porous cryo-crosslinked hydrogel (CCH) embedded with H₂TiO₃ ion sieve | Carbon black–loaded hydrogel (interfacial solar evaporation) | Solar evaporation–driven ion pumping coupled with Li⁺ ion-sieve adsorption (HTO) | 24.7 mg/g (real salt-lake brine); ~27.7 mg/g (simulated brine) | High Li⁺ selectivity over Mg²⁺ (Li⁺/Mg²⁺ up to ~413); directional salt crystallization | High selectivity, zero-liquid discharge, integrated freshwater generation; system complexity and reliance on structured evaporator design |
| Ding et.al. ^12^ | Photothermal-driven hydrogel micromotors (PHF-x) | Fe₃O₄ and HMO nanoparticles embedded in PVA hydrogel | Solar-driven thermophoretic motion coupled with HMO ion-sieve adsorption | 34.5 ± 1.1 mg/g (0.1 W/cm², 1.5 h, 50 ppm Li⁺) | Very high Li⁺/Mg²⁺ selectivity (MLR reduced from 160 to <1) | Fast kinetics and high selectivity; system complexity and reliance on micromotor propulsion |
| This work | Mo-LDH@Sponge | MoS₂ photothermal layer integrated on porous sponge | Hydration-controlled Li⁺ adsorption on Li/Al-LDH regulated by photothermal interfacial heatin | ~150 mg/dm³ under solar illumination (steady-state, 0.67 sun) | High Li⁺ selectivity via hydration shell modulation and LDH chemistry; coupled evaporation–adsorption | Structurally integrated and energy-efficient system; performance influenced by mass transport under high evaporation flux |

1. XPS atomic percentages of the material before and after Li+ adsorption.

**Table S5.** XPS atomic percentages before and after Li⁺ adsorption.

| Element | Before adsorption (at.%) | After adsorption (at.%) |
| --- | --- | --- |
| O 1s | 79.0 | 78.8 |
| Al 2p | 18.1 | 18.0 |
| Cl 2p | 2.9 | 3.2 |
| Li 1s | < 0.1 | < 0.1 |

1. SEM and XRD analysis of pure MoS_2_ and Li/Al-LDH materials.


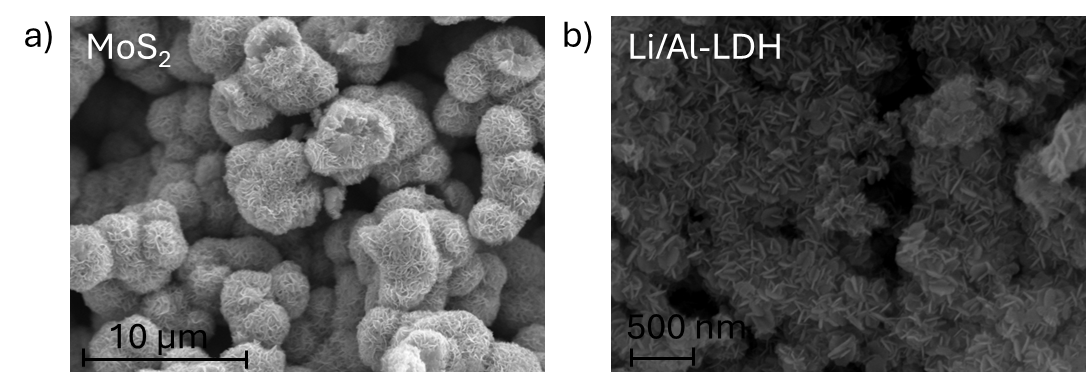


**Figure S5.** SEM images of a) MoS_2_ and b) Li/Al-LDH materials.


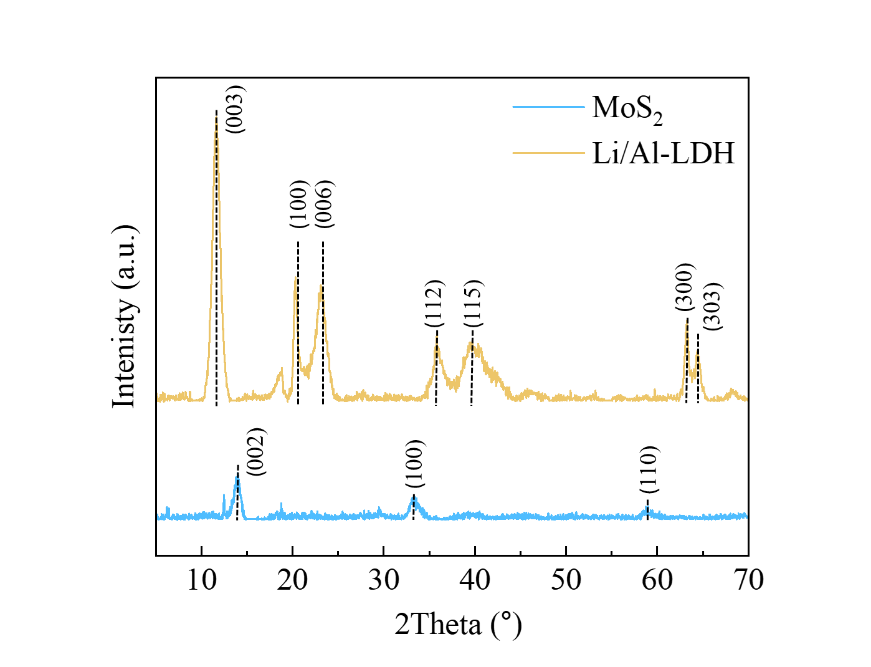


**Figure S6.** XRD spectra of MoS_2_ and Li/Al-LDH materials.

Reference:

(1) Song, Y.; Fang, S.; Xu, N.; Wang, M.; Chen, S.; Chen, J.; Mi, B.; Zhu, J. Solar transpiration–powered lithium extraction and storage. *Science* **2024**, *385* (6716), 1444-1449. DOI: doi:10.1126/science.adm7034.

(2) Xia, Q.; Deng, Z.; Sun, S.; Zhao, W.; Ding, J.; Xi, B.; Gao, G.; Wang, C. Solar-enhanced lithium extraction with self-sustaining water recycling from salt-lake brines. *Proceedings of the National Academy of Sciences* **2024**, *121* (23), e2400159121.

(3) Chen, X.; Yu, W.; Zhang, Y.; Huang, C.; Nie, L.; Yu, J.; Zhang, Y.; Zhang, C.; Zhai, W.; Zhang, X. Solar‐Driven Lithium Extraction by a Floating Felt. *Advanced Functional Materials* **2024**, *34* (28), 2316178.

(4) Zhang, S.; Wei, X.; Cao, X.; Peng, M.; Wang, M.; Jiang, L.; Jin, J. Solar-driven membrane separation for direct lithium extraction from artificial salt-lake brine. *Nature communications* **2024**, *15* (1), 238.

(5) Zhao, Q.; Zhong, H.; Lai, Z.; Ji, Y.; Qin, Q.; Wang, S. Utilizing solar energy for targeted lithium extraction from salt lake brine with laser-induction. *Desalination* **2025**, *599*, 118449.

(6) Zhou, Z.; Wang, H.; Li, Q.; Gao, R.; Feng, K.; Pan, R.; Qu, J.; Gong, J.; Niu, R. Solar-driven pump for simultaneous lithium capture and freshwater generation from salt-lake brine. *Journal of Materials Science & Technology* **2025**.

(7) Li, H.-N.; Zhang, C.; Xin, J.-H.; Liu, Y.-W.; Yang, H.-C.; Zhu, C.-Y.; Liu, C.; Xu, Z.-K. Design of photothermal “ion pumps” for achieving energy-efficient, augmented, and durable lithium extraction from seawater. *ACS nano* **2024**, *18* (3), 2434-2445.

(8) Wang, Z.; Chen, Z.; Li, Y.; Ren, X.; Xiong, X.; Lu, Z.; Deng, L. Photothermal-enhanced ion transport for efficient electrochemical lithium extraction at low temperatures. *Nano Energy* **2024**, *131*, 110249. DOI: <https://doi.org/10.1016/j.nanoen.2024.110249>.

(9) Zhong, H.; Zhao, Q.; Lai, Z.; Xu, D.; Ji, Y.; Wang, S.; Qin, Q. Efficient and Selective Lithium Extraction from Brine Water Via a Photothermal Sandwich Sieve Structure. *Advanced Functional Materials* **2025**, *35* (16), 2418358.

(10) Yu, Z.; Mao, Z.; Guo, S.; Li, Y.; Cheng, X.; Li, C.; Li, L.; Duan, F.; Li, W.; Zhang, Y. Adsorption-responsive bionic photothermal ion pump for reversible seawater lithium extraction. *Nature Communications* **2025**, *16* (1), 8825.

(11) Xing, W.; Wang, D.; Feng, K.; Ding, S.; Zhang, X.; Xu, H.; Gong, J.; Qu, J.; Niu, R. Interfacial solar evaporation-driven lithium extraction from salt-lake brines for battery-grade Li 2 CO 3 production. *Energy & Environmental Science* **2025**, *18* (23), 10102-10111.

(12) Ding, S.; Xing, W.; Chen, L.; Fu, L.; Zhang, X.; Luo, Z.; Li, Q.; Qin, M.; Gong, J.; Qu, J.; et al. Photothermal-driven micromotor for enhanced lithium extraction from salt-lake brines. *Chemical Engineering Journal* **2025**, *522*, 167728. DOI: <https://doi.org/10.1016/j.cej.2025.167728>.
